# Meta-Analysis of MicroRNAs Dysregulated in the Hippocampal Dentate Gyrus of Animal Models of Epilepsy

**DOI:** 10.1523/ENEURO.0152-17.2017

**Published:** 2017-12-28

**Authors:** Prashant K. Srivastava, Paolo Roncon, Katarzyna Lukasiuk, Jan A. Gorter, Eleonora Aronica, Asla Pitkänen, Enrico Petretto, Michael R. Johnson, Michele Simonato

**Affiliations:** 1 Division of Brain Sciences, Imperial College London, Charing Cross Hospital, W12 0NN London, United Kingdom; 2 Division of Neuroscience, University Vita-Salute San Raffaele, Milan 20132, Italy; 3 Nencki Institute of Experimental Biology, Polish Academy of Sciences, 02-093 Warsaw, Poland; 4 Swammerdam Institute for Life Sciences, Center for Neuroscience University of Amsterdam, 1105 Amsterdam, The Netherlands; 5 Department of (Neuro)Pathology, Academic Medical Center and SEIN – Stichting Epilepsie Instellingen Nederland, The Netherlands; 6 Department of Neurobiology, A.I. Virtanen Institute for Molecular Sciences University of Eastern Finland, FIN-70 211 Kuopio, Finland; 7 Duke-NUS Medical School, Singapore 169857, Singapore; 8 Department of Medical Sciences, Section of Pharmacology, University of Ferrara, Ferrara, Italy; 9 National Institute of Neuroscience, Italy; 10 Laboratory for Technologies of Advanced Therapies (LTTA), Ferrara 44121, Italy

**Keywords:** dentate gyrus, epilepsy, hippocampus, meta-analysis, miRNA, mRNA

## Abstract

The identification of mechanisms transforming normal to seizure-generating tissue after brain injury is key to developing new antiepileptogenic treatments. MicroRNAs (miRNAs) may act as regulators and potential treatment targets for epileptogenesis. Here, we undertook a meta-analysis of changes in miRNA expression in the hippocampal dentate gyrus (DG) following an epileptogenic insult in three epilepsy models. We identified 26 miRNAs significantly differentially expressed during epileptogenesis, and five differentially expressed in chronic epilepsy. Of these, 13 were not identified in any of the individual studies. To assess the role of these miRNAs, we predicted their mRNA targets and then filtered the list to include only target genes expressed in DG and negatively correlated with miRNA expression. Functional enrichment analysis of mRNA targets of miRNAs dysregulated during epileptogenesis suggested a role for molecular processes related to inflammation and synaptic function. Our results identify new miRNAs associated with epileptogenesis from existing data, highlighting the utility of meta-analysis in maximizing value from preclinical data.

## Significance Statement

Meta-analyses of data from human research studies are an invaluable tool, and the methods to conduct these investigations are well established. However, meta-analyses of preclinical data are rarely undertaken, due to the typically small sample sizes and the substantial heterogeneity between studies. We implemented a meta-analysis of microRNA (miRNA) expression changes in animal studies of epilepsy. This is the first study of its kind in the field of epilepsy and one of the first in preclinical research. Our analyses identify new miRNAs associated with epileptogenesis and epilepsy, highlighting common mechanisms across different animal models. These miRNAs and their predicted effects on gene expression generate new hypotheses about the causes of epilepsy that will prompt new studies in the field.

## Introduction

Epilepsy is a serious, common neurologic disorder primarily characterized by the occurrence of spontaneous seizures. The treatment of epilepsy remains as one of the major unmet medical needs in neurology because, despite of over 20 antiepileptic drugs on the market, seizures are not controlled in about one third of the patients. The most common form of epilepsy in adults originates in temporal structures of the brain (temporal lobe epilepsy, TLE; Hauser et al., 1993). Epilepsy (TLE in particular) frequently arises as a consequence of brain injury (“acquired epilepsy”). While acquired epilepsies are in principle preventable by the therapeutic targeting of molecular processes underpinning their development (i.e., antiepileptogenic therapies), there are currently no established treatment options for halting the transformation of normal brain tissue to epileptic (Simonato et al., 2014). Identification of the mechanisms underlying epileptogenesis would therefore facilitate the identification of therapies for preventing the development of epilepsy and may inform new strategies for overcoming drug resistance in epilepsy more generally (Simonato et al., 2012; 2013; 2014).

Recent studies have suggested that microRNAs (miRNAs) play an important role in the pathogenesis of acquired epilepsy and may represent novel therapeutic targets (Jimenez-Mateos et al., 2012; Brennan et al., 2016; for review, see Cattani et al., 2016; Henshall et al., 2016). miRNAs are a family of small (21-25 nucleotides) noncoding RNAs, which can modulate various cellular and biological processes by degrading or repressing translation of specific mRNAs (Bartel, 2004, Guo et al., 2010). In systems analysis, miRNAs and their gene targets are described as following a “many-to-many” data model, such that each miRNA may regulate many transcripts and a single transcript may be regulated by many miRNAs (Ebert and Sharp, 2012). miRNAs have been implicated in various neuronal functions that are relevant in the pathogenesis of neurologic diseases, including epilepsy (Tan et al., 2013; Rajman et al., 2017; for review, see Karnati et al., 2015).

Interpretation of data investigating the dysregulation of miRNAs in the brains of patients with epilepsy is challenged by the absence of appropriate human control brain tissue (Roncon et al., 2016). Research on the role of miRNAs in epilepsy has therefore focused on the use of experimental models of epilepsy, revealing changes in the expression of hippocampal miRNAs at different stages of the epileptogenic process (Henshall et al., 2016). However, methodological differences between the various preclinical animal models of epilepsy have made comparisons between studies difficult and the identification of common pathways dysregulated in epileptogenesis and epilepsy problematic. The current preclinical miRNA studies vary in multiple parameters, including brain region analyzed, animal model, sample size, microarray platform and analysis technique. Moreover, these studies are generally substantially underpowered to reliably detect modest changes in miRNA expression.

One particular concern is that analysis of large brain regions (like the hippocampus or the cortex) across different studies may confound interpretation and comparison because of variable cellular composition (e.g., relating to variable neuronal loss, astrocytosis, microgliosis, etc.). One way to address this issue could be to focus on a specific cell population. In this respect, dentate gyrus (DG) granule cells (GCs) seem particularly attractive as a target of analysis as the DG GC layer is a compact layer of (almost) identical cells, facilitating the dissection of a nearly pure cell population (GCs). Moreover, the DG has been traditionally described as a “gate” to inhibit hippocampal overexcitation (Chevaleyre and Siegelbaum, 2010) and recently, this hypothesis found support from new technologies; optogenetic GC hyperpolarization was found to stop spontaneous seizures, whereas optogenetic GC activation exacerbated spontaneous seizures, and activating GCs in nonepileptic animals evoked acute seizures (Krook-Magnuson et al., 2015). Finally, the DG is known to undergo important functional changes during epileptogenesis (neurogenesis, mossy fiber sprouting, increased excitation; Pitkänen and Lukasiuk, 2011).

To date, three studies have investigated differential expression of miRNAs in the DG during the epileptogenesis and the chronic phase of epilepsy in rats (Bot et al., 2013; Gorter et al., 2014; Roncon et al., 2015). Each of these studies used a different method to trigger epileptogenesis: focal electrical stimulation of the lateral nucleus of the amygdala (Bot et al., 2013); focal electrical stimulation of the angular bundle, a major afferent pathway from the entorhinal cortex to the hippocampus (Gorter et al., 2014); and the systemically administered chemoconvulsant pilocarpine (Roncon et al., 2015). Interestingly, all these models imply a key involvement of the DG in the development of epilepsy but via a different epileptogenic insult: direct activation in the case of angular bundle stimulation, indirect in amygdala stimulation, and a widespread brain activation in the case of pilocarpine (Peng and Houser, 2005).

Here, we aimed to overcome some of the limitations related to individual studies by combining these three studies in a meta-analysis, the aim being to increase the statistical power for detecting differentially expressed miRNAs while accounting for study heterogeneity, ultimately leading to more robust and accurate predictions of dysregulated downstream pathways (Ramasamy et al., 2008; Yang et al., 2014). Moreover, this approach offers the opportunity to identify miRNA changes that are independent of the model of epilepsy, i.e., more likely to be disease rather than model related. Our meta-analysis was performed at two time points in the “natural history” of the experimental disease: epileptogenesis and the chronic phase of epilepsy.

## Materials and Methods

### Inclusion criteria and study design

We collected datasets for meta-analysis based on available genome-wide expression profiles of miRNAs from the DG from epileptic and control hippocampi during epileptogenesis and chronic epilepsy. To assist the functional inference of differentially expressed miRNAs we analyzed publicly available gene expression data obtained from the DG of epileptic rodents.

To identify relevant studies, we first undertook a systematic search to identify all published studies of miRNA expression levels and/or gene expression between cases (epileptic) and controls, in the DG of animal models of epilepsy. We conducted a PubMed search based on the string: “(microRNA OR miRNA) AND (dentate gyrus OR dentate cells OR granule cells) AND epilepsy.” miRNAs or genes expression profiles data obtained from the whole hippocampi or different brain regions to the DG were not included in the meta-analysis. This search and inclusion criteria identified only three relevant articles (Bot et al., 2013; Gorter et al., 2014; Roncon et al., 2015).

Time points for each stage of epileptogenesis and for the chronic phase of epilepsy have not been standardized. For the purposes of this study, we considered 7-8 d after SE as the “epileptogenesis phase” and more than two months after SE as the “chronic phase.”

The following models were used in the three relevant papers and considered for the meta-analysis. (1) Pilocarpine model: a microarray study based on the investigation of miRNAs differentially expressed in the laser-microdissected GC layer of the DG of the hippocampi of pilocarpine-induced epileptic rats and matched controls (*n* = 4), killed during the late phase of latency, 8 d after SE (*n* = 5) and in the chronic stage, 50 d after the first spontaneous seizure (*n* = 5; Roncon et al., 2015). (2) Amygdala stimulation: a microarray study focused on the differential expression of miRNAs and genes in hand-dissected DG in the amygdala stimulation rat model during the phase of epileptogenesis, 7 d after the stimulation (*n* = 5), in the chronic stage, 60 d after the stimulation (*n* = 5) and controls (*n* = 5; Bot et al., 2013). (3) Perforant path stimulation: a microarray miRNA study based on the perforant path stimulation rat model of epilepsy. We analyzed DG samples obtained from stimulated and control rat hippocampi, during latency (8 d after SE; *n* = 8) and three to four months after the stimulation for the chronic stage (*n* = 6) and controls (*n* = 10; Gorter et al., 2014). The datasets considered for the meta-analysis are summarized in Table 1.

**Table 1.** Datasets included in the meta-analysis

	Roncon et al. (2015)	Bot et al. (2013)	Gorter et al. (2014)	Dingledine et al. (2017)	Dingledine et al. (2017)	Dingledine et al. (2017)
GEO ID	**-**	GSE49849	**-**	GSE47752	GSE47752	GSE47752
Rat model	Pilocarpine	Amygdala stimulation	Angular bundle stimulation	Pilocarpine	SSSE	Kainate
Sample count epileptogenesiscases:control	5:5	5:5	8:10	6:6	4:5	6:6
Sample count chronic stagecases:control	5:4	5:5	6:10	**-**	**-**	**-**
Platform	Rat miRNA MicroArray kit, Agilent Technologies	miRCURY LNA microRNA Array7th, Exiqon services	miRCURY LNA microRNA Array 6th, Exiqon services	GeneChip Rat Genome 230 2.0 Array, Affymetrix	GeneChip Rat Genome 230 2.0 Array, Affymetrix	GeneChip Rat Genome 230 2.0 Array, Affymetrix
miRNA/gene expression data	miRNA	miRNA and gene expression	miRNA	Gene expression	Gene expression	Gene expression
Tissue collection	Laser-microdissected DG	Handily dissected DG	Handily dissected DG	Laser-microdissected DG	Laser-microdissected DG	Laser-microdissected DG

### Power calculation

**Figure 1.**
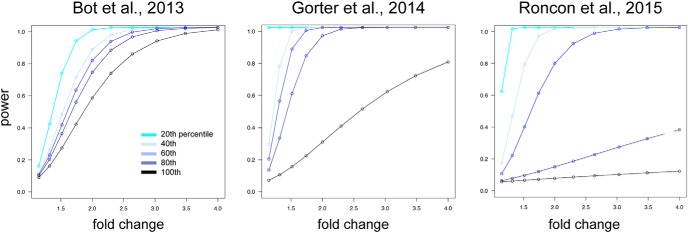
Power calculation. Power calculation is plotted as the power (*y*-axis) to detect a miRNA with fold change (*x*-axis) according to the percentile of the ranked SDs for miRNAs for each study. Across all three models, the power to detect miRNA with fold change 2 or less falls below 80% for at least 40% of the miRNAs.

The statistical power of cases and controls for each individual model was calculated using pooled SD of each expressed miRNA. Power to detect miRNAs differentially expressed at multiple fold changes (1, 1.5, 2, 2.5, 3, 3.5, and 4) were calculated, considering miRNA expression variability ranging from 20th, 40th, 60th, and 80th to 100th percentile of the respective SD profiles per model (Fig. 1). Power calculations were performed using R bioconductor package ‘pwr’ version 1.2.

### Data processing

From each identified study the following information was extracted: platform, number of cases and controls, and miRNA expression data at different time points of the disease. When available, GEO accession number and gene expression data were extracted (Table 1).

#### Data transformation

Since different platforms had been used to generate miRNA expression values, a linear transformation approach was applied to each miRNA using a Z-score transformation according to the formula:

$$Z–score=\frac{Xi-X}{\delta }$$

Where Xi is the normalized intensity data for each miRNA, X is the average normalized miRNA intensity within a single study, and δ is the SD of cases and controls within respective studies.

#### Effect size estimation

A meta-analysis is “a technique for quantitatively combining and integrating the results of multiple studies on a given topic” (Polit and Beck, 2004). Thus, a key aspect of meta-analysis is to measure differences and direction of change from quantitative research studies (Polit and Beck, 2004; Berben et al., 2012). A common metric used to provide this important information is the effect size calculation. Accordingly, to give a statistical expression of the magnitude of the difference between groups (i.e., epileptogenesis vs controls and chronic stage vs controls) in regard of miRNAs expression, we estimated the effect size of each individual study defined as the standardized mean difference (SMD) between cases and controls. The SMD has been calculated using the Hedge’s method with the following formula:

$$g=\frac{X_{1}-X_{2}}{\delta }$$

Where X_1_ is the mean of cases, X_2_ the mean of the control group, and δ is the SD.

#### Statistical heterogeneity

Different animal models, tissue collection methods, and platforms were used to generate the datasets. This makes it difficult to directly compare the data, the risk being of skewing comparison results, reducing the reliability of measurements of individual miRNA expression changes (Yang et al., 2014). Statistical heterogeneity was assessed using Cochrane meta-regression approach calculated by Q test, I^2^ statistics, and Tau^2^ statistics. These measures were applied at each dataset to assess the overall heterogeneity (Higgins et al., 2003; Ioannidis et al., 2007). To test the total variance of each miRNA within the studies, the Cochran Q test have been run, according to the formula:

$$Q=k(k-1)\frac{\overset{k}{\underset{T-1}{\sum }}{\left(xT-\frac{N}{k}\right)}^{2}}{\overset{b}{\underset{i-1}{\sum }}xT(k-xT)}$$

Where k is the number of the studies included in the meta-analysis, T is the number of variables observed, b is the number of miRNAs included in the test, and N is the total number. A Benjamini-Hochberg (BH) adjusted *p* < 0.05 was considered statistically significant for the Cochrane Q test.

Furthermore, I^2^ statistic has been employed to describe the percentage of the variability in the effect size estimates, following the formula:

$$I^{2}=\left(\frac{Q-df}{Q}\right)*100\%$$

Where Q is the value derived from the Cochran Q test and df are the degrees of freedom. Tentatively, I^2^ statistic can be considered as an indicator of heterogeneity, where low, moderate, and high heterogeneity corresponds to I^2^ values of 0–0.3, 0.3–0.7, and 0.7–1, respectively (Higgins et al., 2003).

Finally, to estimate the variance across studies the Tau^2^ has been applied:

$$Tau^{2}=\frac{Q-df}{C}$$

Where Q is the value derived from the Cochran Q test, df are the degrees of freedom and C is a scaling factor which takes into consideration that the Q value is the weighted sum of squares.

#### Meta-analysis

The presence of statistical heterogeneity among the studies led us to use a random-effect model for the meta-analysis rather than a fixed-effect model. The pooled effect size (PES) for each miRNA was obtained applying the random effect size model based on the DerSimonian and Laird method (DerSimonian and Laird, 1986; DerSimonian and Kacker, 2007). We generated one forest plot for each miRNA in both the epileptogenesis and the chronic phase to depict the SMD along with its 95% confidence interval (95% CI) for individual studies as well as the pooled MD from the meta-analysis.

### miRNAs:mRNAs inverse-fold change

miRNAs predominantly act by repressing their target genes by decreasing target mRNA levels (Bartel, 2004; Guo et al., 2010). Therefore, we investigated the correlation of mRNAs predicted targets expression in the available transcriptomic datasets.

To predict miRNA target genes, the miRNA-target interactions were analyzed with the web-based tool miRwalk (Dweep et al., 2011; Dweep et al., 2014). The rule that the 5’ region of miRNA from nucleotides 2-8 (“seed region”) has importance in targeting, is commonly accepted as the canonical mechanism by which miRNAs complementary convey functional binding to mRNA targets (Jinek and Doudna, 2009). However, despite the importance of the seed region, the 3’ end of a mRNA also contributes to the binding in ∼2% of all preferentially conserved sites (Grimson et al., 2007; Shkumatava et al., 2009). In addition, some validated miRNAs have a binding site that exhibits few continuous base pairs in the control region (Shin et al., 2010). Thus, to figure out the complete mechanism of miRNA regulation, we expanded the miRNAs-binding site prediction within the 3’, 5’ untranslated regions (UTRs), and the seed sequence, with a minimum seed length of seven nucleotides. Furthermore, to exclude overprediction we applied a comparative analysis by six prediction programs: miRMap, RNA22, miRanda, RNAhybrid, PICTAR2, and Targetscan. Following this approach, a candidate mRNA target has to be identified by all these programs. As we did for the miRNAs, we conducted PubMed search based on the keywords: “gene expression, epilepsy, dentate gyrus.” Two studies were identified that were then included in our analysis (Table 1): Dingledine et al. (2017; GEO repository accession number: GSE47752) and Bot et al. (2013; GEO repository accession number: GSE49850). The first includes gene expression data obtained from laser-microdissected DG of rats that received different SE models of epilepsy: pilocarpine, self-sustained SE (SSSE), and kainite, it also included kindling, not considered in this study not being a post-SE model. We considered in our analysis only those rats killed 10 d after SE that did not develop spontaneous seizures, as the best time point matching the epileptogenesis phase used for the miRNAs meta-analysis (7-8 d after SE). In Dingledine et al. (2017), the kainate and pilocarpine models were performed in two independent labs, while the SSSE model that was performed in only one lab. For studies undertaken in multiple labs, we combined the *p* values (obtained from differential expression analysis) from the independent labs using Fisher’s method. The second dataset (GSE49850) was obtained from the same amygdala-stimulated rats used for the miRNA analysis.

To investigate miRNA-mRNA interactions, we included only those mRNAs that had inverse relationship to miRNA changes in at least three datasets in the epileptogenesis phase; while for the chronic phase, we considered all predicted mRNAs that presented an inverse relationship to miRNA changes in the amygdala stimulation dataset only.

### miRNA functional enrichment analysis

Functional enrichment analysis using gene ontology (GO) and pathways enrichment analysis based on the Kyoto encyclopedia of genes and genomes (KEGG) database were performed using Webgestalt webserver (Zhang et al., 2005; Wang et al., 2013). The enrichment was performed with a hypergeometric test separately for the list of predicted targets based on those miRNAs dysregulated in epileptogenesis and in the chronic stage. Significant canonical pathways maps were selected according to a false discovery rate (FDR) < 5%.

To infer functional relationships between miRNAs identified using meta-analysis, a network of miRNAs based on their ability to target common pathways has been generated. A connection was made between a pair of miRNA, if respective mRNA targets belonged to the same pathways or GO terms that were significantly (FDR < 0.05) enriched for combined miRNA targets. The network was visualizes using Cytoscape version 2.8.2.

## Results

### Study design

**Figure 2.**
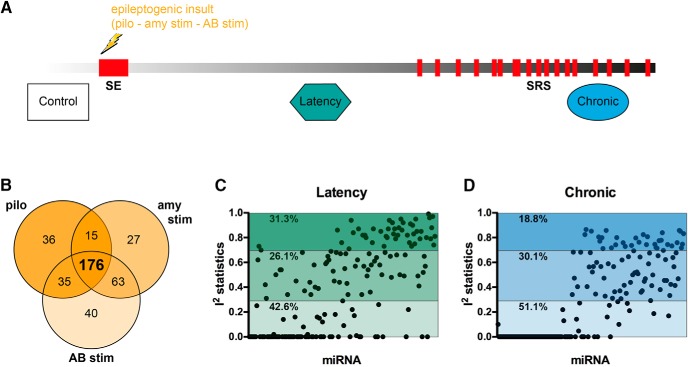
Study design and data preprocessing. ***A***, Study design. ***B***, Venn diagram showing miRNAs commonly expressed between the three studies included in the meta-analysis. ***C***, Statistical heterogeneity estimation. I^2^ scores of commonly expressed miRNAs (*n* = 176) in epileptogenesis and in chronic stage. miRNAs are ordered based on the adjusted *p* value after meta-analysis. I^2^ < 0.3, low heterogeneity; 0.3 < I^2^ > 0.7, moderate heterogeneity; I^2^ > 0.7, high heterogeneity. SRS, spontaneous recurrent seizures; pilo, pilocarpine model; amy stim, amygdala stimulation model; AB stim, angular bundle stimulation model.

We included in the meta-analysis all published miRNA expression datasets from dissected DG of the hippocampus that compared control (baseline) tissue with tissue from epileptogenesis and chronically epileptic rats (Fig. 2*A*). Based on these inclusion criteria, we identified three datasets that used different epilepsy models (Table 1): (1) pilocarpine (Roncon et al., 2015), (2) amygdala stimulation (Bot et al., 2013), (3) angular bundle stimulation (Gorter et al., 2014). We first calculated the power of each individual study to detect significant changes in miRNA expression and found that all three individual studies were substantially underpowered to detect modest fold changes (<2.0) in miRNA expression (Fig. 1). Of the total number of miRNAs expressed at any time point in any of the three models, the expression levels of 176 miRNAs were detectable across all three studies (Fig. 2*B*). The expression values of these 176 miRNAs were then Z-score transformed and meta-analyzed across the three studies.

### Estimation of statistical heterogeneity

Meta-regression analyses were performed separately for epileptogenesis and chronic stages of epilepsy for the 176 miRNAs that were expressed in all three datasets (Fig. 2*B*). There are two models that are commonly used to perform meta-analysis, the fixed effect and the random effects models. The fixed effect model assumes that the effect size is the same in all studies, while the random effects analysis assumes that the effect can vary from one study to another. To determine the correct model for this study, we first estimated statistical heterogeneity using the Cochrane’s Q test, separately for the epileptogenesis and the chronic phase. This revealed significant heterogeneity between studies at both stages of the disease (BH, adjusted *p* < 0.05). Next, to assess the proportion of miRNAs that were differentially expressed between studies, during epileptogenesis and the chronic stage separately, we calculated the I^2^ statistics (Higgins et al., 2003). Of the 176 miRNAs measured across the three studies, 26.14% revealed a high level of heterogeneity: 31.25% a moderate level and 42.61% a low rate of heterogeneity during epileptogenesis (Fig. 2*C*). In the chronic stage, 18.75% displayed high, 30.11% moderate, and 51.14% low level heterogeneity (Fig. 2*C*). Collectively, these observations favor the use of the random effects model.

### Differential expression of miRNAs in the epileptic DG

**Figure 3.**
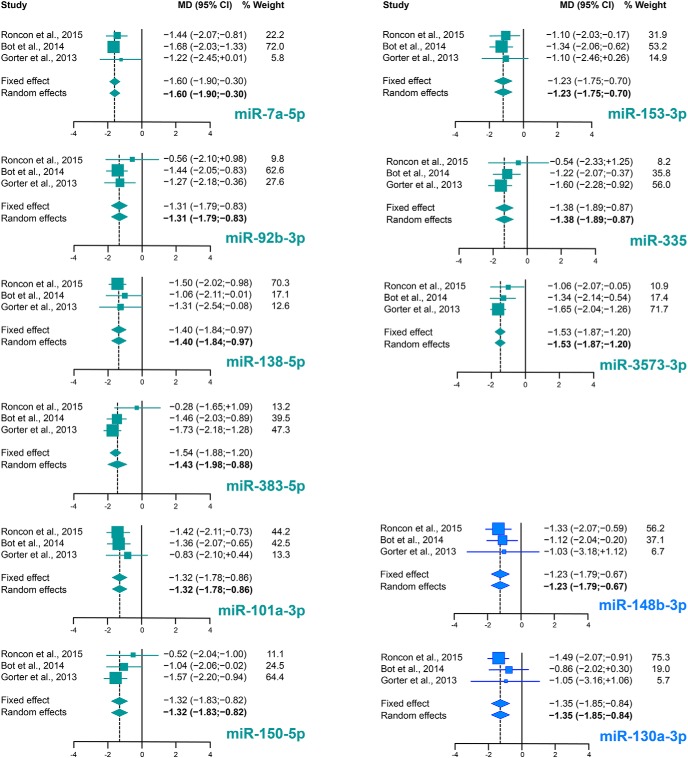
Forest plots of selected miRNA. Forest plots for miR-7a-5p, miR-92b-3p, miR-101a-3p, miR-138-5p, miR-150-3p, miR-153-3p, miR-335, miR-383-3p, and miR-3573-3p are shown for the phase of epileptogenesis, and miR-130a-3p and miR-148b-3p for the chronic stage. For each miRNA, the effect size of the individual studies is reported as MD and 95% CI. The % weight refers to random effects analysis. Individual effect sizes are represented by colored boxes (green for epileptogenesis and blue for the chronic period) and 95% CI are denoted by black lines. The combined effect sizes are represented by diamonds, where diamond width correspond to the 95% CI bounds; boxes and diamonds size is proportional to effect size estimation precision. For each miRNA, the weight of the dataset in the combined analysis has been reported in percentage.

Using a random effects meta-analysis of miRNA changes in the three models of epileptogenesis and adopting a stringent correction for multiple testing to minimize false positives (Bonferroni adjusted *p* < 0.05), we identified 26 and 5 differentially expressed miRNAs between control and latency and between control and chronic epilepsy, respectively. Full results of all these miRNAs including PES estimations, I^2^, Tau^2^ and *p* values are shown in Tables 2, 3. Forest plots for selected miRNA are shown in Figure 3. Comparing these results with those presented in each original studies that have been meta-analyzed here, our meta-analysis identified 11 miRNAs differentially expressed in epileptogenesis compared to control and two miRNAs (i.e., miR-324-3p and miR-130a-3p) in the chronic stage of epilepsy that were not identified as significantly differentially expressed in any of the individual studies (Tables 2, 3, miRNAs highlighted in bold). The datasets employed in this meta-analysis do not allow a precise evaluation of the abundance of expression of these 26 plus five miRNAs under control conditions, but a relative abundance estimate based on the internal standards employed in each study suggests that almost all are expressed at medium to high abundance in the control DG (only miR-212-5p was expressed at relatively low levels but upregulated during epileptogenesis).

**Table 2.** Differentially expressed microRNAs in the epileptogenesis period compared with controls

miRNA	ESestimation	Nominal*p* value	Bonferroni adjusted*p* value	*Q* statistics	I^2^ statistics	Tau^2^ statistics
*miR-212-3p*	1.70	6.21^−16^	1.10^−13^	1.59	0.00	0.00
miR-7a-5p	-1.60	9.26^−12^	1.64^−09^	0.82	0.00	0.00
miR-33-5p	-1.57	9.90^−12^	1.75^−09^	0.22	0.00	0.00
miR-139-5p	-1.53	8.34^−11^	1.48^−08^	0.29	0.00	0.00
*miR-344b-2-3p*	1.25	5.78^−10^	1.02^−07^	1.57	0.00	0.00
**miR-3573-3p**	-1.53	1.32^−09^	2.33^−07^	1.38	0.00	0.00
miR-551b-3p	-1.51	2.16^−09^	3.83^−07^	0.51	0.00	0.00
*miR-146a-5p*	1.75	3.00^−09^	5.31^−07^	2.66	0.25	0.25
*miR-132-3p*	1.85	1.46^−08^	2.58^−06^	5.28	0.62	0.24
**let-7b-3p**	-1.34	2.46^−07^	4.36^−05^	7.40	0.73	0.42
*miR-212-5p*	1.36	4.58^−07^	8.11^−05^	0.36	0.00	0.00
**let-7d-3p**	-1.42	9.57^−07^	0.0002	6.75	0.70	0.38
**miR-667-3p**	-1.37	1.01^−06^	0.0002	1.87	0.00	0.00
miR-138-5p	-1.40	1.34^−06^	0.0002	0.59	0.00	0.00
miR-330-3p	-1.37	1.39^−06^	0.0002	0.17	0.00	0.00
*miR-21-5p*	1.85	2.65^−06^	0.0005	3.43	0.42	0.17
miR-29c-5p	-1.65	5.76^−06^	0.0010	2.91	0.31	0.27
**miR-335**	-1.38	6.01^−06^	0.0010	1.41	0.00	0.00
**miR-101a-3p**	-1.32	9.17^−06^	0.0016	0.64	0.00	0.00
**miR-345-5p**	-1.29	2.12^−05^	0.0037	1.75	0.00	0.00
miR-92b-3p	-1.31	3.12^−05^	0.0055	1.08	0.00	0.00
**miR-150-5p**	-1.32	3.46^−05^	0.0061	1.94	0.00	0.00
**miR-136-3p**	-1.22	3.56^−05^	0.0063	0.66	0.00	0.00
miR-324-5p	-1.31	4.63^−05^	0.0082	2.05	0.02	0.08
**miR-153-3p**	-1.23	6.92^−05^	0.0122	0.21	0.00	0.00
**miR-383-5p**	-1.54	0.0002	0.0375	4.00	0.50	0.34

miRNAs in italics are upregulated and miRNAs highlighted in bold were not differentially expressed in individual studies. ES, effect size.

**Table 3.** Differentially expressed microRNAs in the chronic stage compared with controls

miRNA	ES estimation	Nominal*p* value	Bonferroni adjusted*p* value	*Q* statistics	I^2^ statistics	Tau^2^ statistics
miR-652-3p	-1.56	5.48^−09^	9.65^−07^	2.1	0.1	0.1
miR-551b-3p	-1.52	3.20^−06^	0.0006	1.4	0.0	0.0
**miR-324-3p**	-1.40	3.13^−05^	0.0055	0.9	0.0	0.0
**miR-130a-3p**	-1.35	6.10^−05^	0.0107	0.8	0.0	0.0
miR-148b-3p	-1.56	0.0002	0.0317	0.2	0.0	0.0

All miRNAs are downregulated, miRNAs highlighted in bold were not differentially expressed in individual studies. ES, effect size.

### Relationship between miRNAs and mRNAs expression changes

Previous transcriptomic studies in epilepsy models have revealed dysregulation of many genes in the different phases of the experimental disease (Lukasiuk and Pitkänen, 2004). It can be hypothesized that differentially expressed miRNAs may contribute to these alterations (upregulated miRNAs may downregulate their mRNA targets whereas downregulation of miRNAs may allow upregulation of their mRNA targets). To assess the role of differentially expressed miRNAs during the course of epilepsy, that is, to infer their mRNA regulatory targets, we explored the potential regulatory relationship between miRNA and mRNA changes.

First, to predict miRNA target transcripts we used the miRWalk database (Dweep et al., 2011; Dweep et al., 2014), which combines information across six miRNA target prediction programs (miRMap, RNA22, miRanda, RNAhybrid, PITCAR2 and Targetscan). As expected, this analysis identified a very large number of predicted targets but, obviously, the large majority of these may not be expressed in the DG or may not be expressed in a negatively correlated fashion relative to the miRNAs. Therefore, we asked whether the miRNAs that were identified by the meta-analysis as dysregulated in epileptogenesis and in the chronic stage were negatively correlated with changes in DG expression in their predicted mRNA targets. To this end, we took advantage of publicly available gene expression data generated in three separate datasets that investigated mRNA expression changes in the DG of rats from the epilepsy models used in our meta-analysis: pilocarpine (GEO accession: GSE47752; Dingledine et al., 2017), angular bundle stimulation (called SSSE in this database; GEO accession: GSE47752), and amygdala stimulation (Bot et al., 2013; PMID: 24146813). Only the amygdala stimulation mRNA dataset (Bot et al., 2013) was obtained from the same animals employed for obtaining the miRNA dataset and included data on the chronic phase. The other datasets were generated by separate research groups on a separate group of animals, the experimental procedures were slightly different from those used in the corresponding miRNAs studies (Gorter et al., 2014; Roncon et al., 2015) and included mRNA data for these models related to the epileptogenesis phase only. In addition, Dingledine et al. (2017) also included datasets for another SE model (kainate) that we also considered in our analysis.

**Figure 4.**
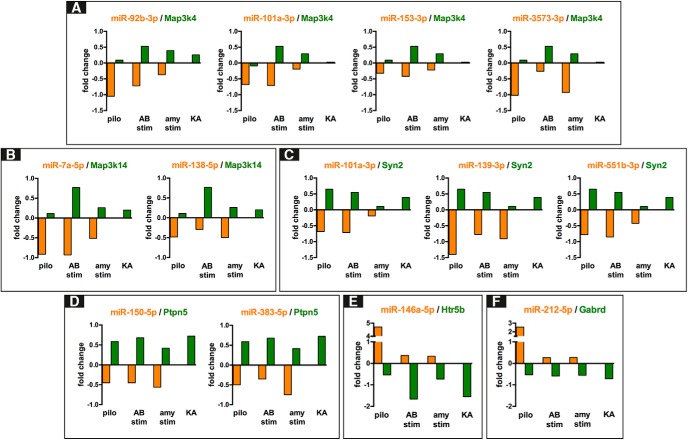
Relationship between selected miRNA and their predicted targets in different model of TLE. All panels show selected miRNAs-mRNA anticorrelation based on miRNAs and mRNAs fold changes in epileptogenesis. ***A***, Inverse relationship between four downregulated miRNAs (miR-92b-3p, miR-101a-3p, miR-153-3p, and miR-3573-3p) and the commonly predicted target *Map3k4*. ***B***, Inverse relationship between the downregulated miR-138-5p, miR-7a-5p, and the upregulated *Map3k14*. ***C***, Inverse relationship between miR-101a-3p, miR-139-3p, miR-551b-3p, and *Syn2*. ***D***, Inverse relationship between miR-150-5p, miR-383-5p, and *Ptpn*. ***E***, ***F***, Examples of the opposite anticorrelation, the upregulated miR-146a-5p with the downregulated *Htr5b* transcript, and the upregulated miR-212-5p and the downregulated *Gabrd* transcript.

Analysis of the epileptogenesis data identified inverse relationship, based on significant fold changes (gene FDR < 0.1), between 22 (of 26) miRNAs and 122 unique predicted gene targets in at least three of the four epilepsy models of the Dingledine dataset (Dingledine et al., 2017) that we considered in this study (Table 4). The mRNAs encoding the mitogen-activated protein kinase kinase kinase 4 (*Map3k4*) and the enhancer of zeste 2 polycomb repressive complex 2 subunit (*Ezh2*) were predicted targets and had inverse expression relative to four miRNAs, i.e., miR-92b-3p, miR-101a-3p, miR-153-3p, and miR-3575-3p for *Map3k4* and miR-92b-3p, miR-101a-3p, miR-138-5p, and miR-153-3p for *Ezh2*; synapsin type 2 was inversely correlated to three miRNAs (miR-101a-3p, miR-139-5p, and miR-551b-3p); the mitogen-activated protein kinase kinase kinase 14 (*Map3k14*) and the protein tyrosine phosphatase, nonreceptor type 5 (*Ptpn5*) were inversely correlated with two miRNAs, i.e., miR-7a-5p and miR-138-5p for *Map3k14*; miR-150-5p and miR-383-5p for *Ptpn5*. All the above transcripts were upregulated during epileptogenesis and predicted targets of miRNAs that were downregulated. In contrast, miR-132-3p, miR-146a-5p, miR-212-3p, and miR-212-5p were upregulated during epileptogenesis. The 5-hydroxytryptamine receptor 5 (*Htr5b*) and the β-1,3-galactosyltransferase 5 (*B3galt5*) were predicted targets of miR-146a-5p and were downregulated. The γ-aminobutiric acid receptor subunit δ (*Gabrd*) was a predicted target of and inversely correlated with miR-212-5p. Finally, we observed that miR-344b-2-3p, let-7d-3p, miR-21-5p, miR-29c-5p, and miR-324-5p were not anticorrelated with any of their predicted mRNA targets. Representative graphs for the anticorrelations between miRNAs and predicted mRNA targets are shown in Figure 4.

**Table 4.** miRNA-mRNA fold changes inverse correlation in epileptogenesis

miRNA name	microRNA data				mRNA targets of respective microRNA (FDR < 0.1)								
	Pilocarpine	Perforant path stimulation	Amygdala stimulation		mRNA gene name	Pilocarpine		Perforant path stimulation		Amygdala stimulation		Kainic acid	
	miRNA FC			Meta-analysis *p* value		FC	Adjusted *p* value	FC	Adjusted *p* value	FC	Adjusted *p* value	FC	Adjusted *p* value
miR-383-5p	-0.50	-0.35	-0.75	6.11^−05^	*Rab32*	0.74	0.01	0.66	0.01	0.28	0.03	0.48	0.06
	-0.50	-0.35	-0.75	6.11^−05^	*Cyb561*	1.07	4.62^−05^	0.53	0.02	0.66	1.41^−03^	0.45	0.09
	-0.50	-0.35	-0.75	6.11^−05^	*Stk40*	0.61	0.05	0.58	0.02	0.35	0.04	0.69	0.04
	-0.50	-0.35	-0.75	6.11^−05^	*Ptpn5*	0.59	1.68^−03^	0.68	0.02	0.42	0.03	0.72	4.39^−04^
	-0.50	-0.35	-0.75	6.11^−05^	*Tyms*	0.41	0.01	0.81	0.03	0.27	0.06	1.10	3.74^−04^
	-0.50	-0.35	-0.75	6.11^−05^	*Ugt1a5*	0.18	0.06	0.32	0.08	0.69	7.29^−04^	0.01	0.97
	-0.50	-0.35	-0.75	6.11^−05^	*Aif1*	0.91	0.04	0.54	0.25	0.70	0.03	0.97	0.02
	-0.50	-0.35	-0.75	6.11^−05^	*Smad3*	0.51	0.06	0.19	0.46	0.45	0.03	0.43	0.08
	-0.50	-0.35	-0.75	6.11^−05^	*Rac2*	0.44	0.03	0.39	0.31	0.98	0.01	0.61	0.11
	-0.50	-0.35	-0.75	6.11^−05^	*Lcmt1*	0.29	0.04	0.25	0.25	0.32	0.02	0.14	0.48
	-0.50	-0.35	-0.75	6.11^−05^	*Mtmr11*	0.95	3.62^−04^	1.86	0.01	0.05	0.87	1.13	4.82^−05^
	-0.50	-0.35	-0.75	6.11^−05^	*Nnat*	1.09	0.01	0.86	0.07	0.15	0.59	0.63	0.30
	-0.50	-0.35	-0.75	6.11^−05^	*Casp3*	0.24	0.07	0.40	0.05	0.27	0.37	0.25	0.40
miR-153-3p	-0.32	-0.42	-0.22	7.95^−04^	*Arhgap17*	0.30	0.02	0.88	0.01	0.43	0.03	0.33	0.08
	-0.32	-0.42	-0.22	7.95^−04^	*Mgst1*	0.67	0.07	0.79	0.01	0.69	0.01	0.29	0.67
	-0.32	-0.42	-0.22	7.95^−04^	*Wls*	1.45	7.49^−05^	0.80	0.01	0.57	0.01	1.75	1.52^−05^
	-0.32	-0.42	-0.22	7.95^−04^	*Map3k4*	0.09	0.59	0.53	0.03	0.29	0.07	0.02	1.00
	-0.32	-0.42	-0.22	7.95^−04^	*Ezh2*	0.16	0.27	0.40	0.07	0.31	0.05	0.04	0.78
	-0.32	-0.42	-0.22	7.95^−04^	*Man2b1*	0.29	0.05	0.19	0.63	0.34	0.02	-0.16	0.87
	-0.32	-0.42	-0.22	7.95^−04^	*Zfp521*	1.23	1.92^−04^	0.74	0.01	0.23	0.22	0.59	3.07^−03^
miR-324-5p	-0.09	-0.60	-0.29	8.55^−06^	*Tyrobp*	1.26	1.01^−04^	0.70	0.26	1.40	4.93^−03^	1.31	0.03
	-0.09	-0.60	-0.29	8.55^−06^	*Asph*	0.45	0.08	0.45	0.03	0.17	0.13	0.05	0.90
	-0.09	-0.60	-0.29	8.55^−06^	*Cyb5r4*	0.36	0.02	0.43	0.07	0.14	0.66	0.50	7.35^−04^
miR-150-5p	-0.45	-0.45	-0.56	6.17^−05^	*E2f1*	0.89	1.05^−03^	0.76	0.01	0.45	0.02	1.11	3.99^−05^
	-0.45	-0.45	-0.56	6.17^−05^	*Cyb561*	1.07	4.62^−05^	0.53	0.02	0.66	1.41^−03^	0.45	0.09
	-0.45	-0.45	-0.56	6.17^−05^	*Ppp1r1a*	0.35	0.03	0.58	0.02	0.44	0.05	0.73	0.01
	-0.45	-0.45	-0.56	6.17^−05^	*Ptpn5*	0.59	1.68^−03^	0.68	0.02	0.42	0.03	0.72	4.39^−04^
	-0.45	-0.45	-0.56	6.17^−05^	*Tyms*	0.41	0.01	0.81	0.03	0.27	0.06	1.10	3.74^−04^
	-0.45	-0.45	-0.56	6.17^−05^	*Igsf1*	1.03	0.37	0.89	0.04	0.31	0.05	0.50	0.49
	-0.45	-0.45	-0.56	6.17^−05^	*Me3*	0.73	0.03	0.61	0.07	0.46	1.41^−03^	1.15	4.17^−07^
	-0.45	-0.45	-0.56	6.17^−05^	*Ugt1a5*	0.18	0.06	0.32	0.08	0.69	7.29^−04^	0.01	0.97
	-0.45	-0.45	-0.56	6.17^−05^	*Slc7a14*	0.91	3.46^−04^	0.42	0.09	0.23	0.06	0.35	0.17
	-0.45	-0.45	-0.56	6.17^−05^	*Tmod3*	0.72	0.01	0.61	0.09	0.30	0.10	0.71	0.07
	-0.45	-0.45	-0.56	6.17^−05^	*Tyrobp*	1.26	1.01^−04^	0.70	0.26	1.40	4.93^−03^	1.31	0.03
	-0.45	-0.45	-0.56	6.17^−05^	*Gpnmb*	1.54	3.04^−03^	0.30	0.68	1.45	0.03	0.81	0.22
	-0.45	-0.45	-0.56	6.17^−05^	*Zmiz1*	0.48	2.39^−03^	0.33	0.14	0.41	0.03	0.49	0.03
	-0.45	-0.45	-0.56	6.17^−05^	*Skap2*	0.73	0.09	0.22	0.37	0.59	0.06	0.57	0.06
	-0.45	-0.45	-0.56	6.17^−05^	*Arhgdib*	0.27	0.01	0.48	0.34	0.85	0.03	0.49	0.19
	-0.45	-0.45	-0.56	6.17^−05^	*Ick*	0.17	0.04	0.28	0.12	0.27	0.10	-0.01	0.88
	-0.45	-0.45	-0.56	6.17^−05^	*Tmem140*	0.80	0.06	0.61	0.05	0.07	0.64	-0.25	0.92
	-0.45	-0.45	-0.56	6.17^−05^	*Trh*	1.20	0.01	1.31	0.02	0.99	0.34	2.87	0.01
	-0.45	-0.45	-0.56	6.17^−05^	*Col9a1*	0.41	0.02	0.91	0.04	0.03	0.92	1.28	2.67^−04^
	-0.45	-0.45	-0.56	6.17^−05^	*Arpp21*	0.11	0.07	0.45	0.09	-0.02	0.91	0.63	0.02
miR-92b-3p	-1.05	-0.72	-0.37	1.70^−05^	*Gadd45a*	-0.20	0.16	1.51	0.01	0.79	0.01	1.33	3.91^−04^
	-1.05	-0.72	-0.37	1.70^−05^	*Map3k4*	0.09	0.59	0.53	0.03	0.29	0.07	0.02	1.00
	-1.05	-0.72	-0.37	1.70^−05^	*Kcnh2*	0.60	0.07	0.63	0.04	0.39	0.09	0.26	0.38
	-1.05	-0.72	-0.37	1.70^−05^	*Ezh2*	0.16	0.27	0.40	0.07	0.31	0.05	0.04	0.78
	-1.05	-0.72	-0.37	1.70^−05^	*Wnt10a*	0.58	1.89^−03^	0.81	0.07	0.46	0.01	1.69	0.01
	-1.05	-0.72	-0.37	1.70^−05^	*Zmiz1*	0.48	2.39^−03^	0.33	0.14	0.41	0.03	0.49	0.03
	-1.05	-0.72	-0.37	1.70^−05^	*Ick*	0.17	0.04	0.28	0.12	0.27	0.10	-0.01	0.88
	-1.05	-0.72	-0.37	1.70^−05^	*Zfp521*	1.23	1.92^−04^	0.74	0.01	0.23	0.22	0.59	3.07^−03^
miR-345-5p	-0.23	-0.25	-0.12	1.46^−06^	*Inpp4b*	0.80	0.10	1.15	2.69^−03^	0.31	0.07	0.24	0.13
	-0.23	-0.25	-0.12	1.46^−06^	*Arhgap17*	0.30	0.02	0.88	0.01	0.43	0.03	0.33	0.08
	-0.23	-0.25	-0.12	1.46^−06^	*Gadd45a*	-0.20	0.16	1.51	0.01	0.79	0.01	1.33	3.91^−04^
	-0.23	-0.25	-0.12	1.46^−06^	*Gcnt1*	1.45	3.63^−05^	1.61	0.06	0.48	0.01	1.87	4.39^−04^
	-0.23	-0.25	-0.12	1.46^−06^	*LOC500956*	0.34	0.20	0.50	0.07	0.27	0.02	0.12	0.91
	-0.23	-0.25	-0.12	1.46^−06^	*Cd74*	1.10	0.04	1.27	0.07	2.81	0.01	1.24	0.11
	-0.23	-0.25	-0.12	1.46^−06^	*Wnt10a*	0.58	1.89^−03^	0.81	0.07	0.46	0.01	1.69	0.01
	-0.23	-0.25	-0.12	1.46^−06^	*Skap2*	0.73	0.09	0.22	0.37	0.59	0.06	0.57	0.06
	-0.23	-0.25	-0.12	1.46^−06^	*Ss18*	0.41	0.02	0.51	0.12	0.58	0.02	0.76	3.58^−04^
	-0.23	-0.25	-0.12	1.46^−06^	*Slfn13*	0.46	0.04	0.41	0.18	1.53	2.69^−03^	0.40	0.12
	-0.23	-0.25	-0.12	1.46^−06^	*Rnd3*	0.87	0.01	0.60	0.07	0.13	0.56	0.36	0.13
	-0.23	-0.25	-0.12	1.46^−06^	*Tmem140*	0.80	0.06	0.61	0.05	0.07	0.64	-0.25	0.92
miR-101a-3p	-0.68	-0.71	-0.19	5.15^−06^	*Map3k4*	0.09	0.59	0.53	0.03	0.29	0.07	0.02	1.00
	-0.68	-0.71	-0.19	5.15^−06^	*Rin2*	0.57	0.06	0.43	0.05	0.44	0.03	-0.06	0.95

	-0.68	-0.71	-0.19	5.15^−06^	*Ezh2*	0.16	0.27	0.40	0.07	0.31	0.05	0.04	0.78
	-0.68	-0.71	-0.19	5.15^−06^	*Arl4a*	0.65	0.02	0.36	0.41	0.40	0.01	0.38	0.09
	-0.68	-0.71	-0.19	5.15^−06^	*Syn2*	0.65	0.04	0.55	0.03	0.11	0.21	0.39	0.03
	-0.68	-0.71	-0.19	5.15^−06^	*Nabp1*	0.56	0.01	0.81	0.01	0.45	0.11	0.96	0.10
	-0.68	-0.71	-0.19	5.15^−06^	*Arpp21*	0.11	0.07	0.45	0.09	-0.02	0.91	0.63	0.02
miR-335	-1.98	-0.39	-0.71	2.47^−05^	*Efr3a*	1.28	0.01	1.40	5.22^−04^	0.49	0.01	1.11	4.17^−07^
	-1.98	-0.39	-0.71	2.47^−05^	*Rprm*	2.68	3.86^−05^	2.54	8.57^−04^	1.00	0.01	1.53	5.05^−04^
	-1.98	-0.39	-0.71	2.47^−05^	*Ackr3*	0.90	0.39	2.38	2.69^−03^	0.55	3.05^−03^	0.90	0.11
	-1.98	-0.39	-0.71	2.47^−05^	*Gcnt1*	1.45	3.63^−05^	1.61	0.06	0.48	0.01	1.87	4.39^−04^
	-1.98	-0.39	-0.71	2.47^−05^	*Fcgr2b*	1.87	3.62^−04^	0.99	0.20	1.52	0.01	1.39	0.01
	-1.98	-0.39	-0.71	2.47^−05^	*Vim*	1.74	0.01	1.01	0.28	0.72	0.05	0.59	0.88
	-1.98	-0.39	-0.71	2.47^−05^	*Arl11*	0.86	0.01	0.08	0.75	0.72	0.01	0.71	0.07
	-1.98	-0.39	-0.71	2.47^−05^	*Epsti1*	0.92	0.03	0.47	0.35	0.71	0.02	0.61	0.02
	-1.98	-0.39	-0.71	2.47^−05^	*Pycard*	0.87	0.01	0.47	0.13	0.47	0.09	0.84	0.01
miR-29c-5p	-0.59	-0.41	-0.14	1.48^−07^	*E2f1*	0.89	1.05^−03^	0.76	0.01	0.45	0.02	1.11	3.99^−05^
	-0.59	-0.41	-0.14	1.48^−07^	*Tmem176b*	0.75	0.48	0.67	0.05	0.97	0.01	1.12	0.02
	-0.59	-0.41	-0.14	1.48^−07^	*Sh3bgrl3*	0.77	0.01	0.49	0.13	0.48	4.00^−03^	0.70	0.01
miR-330-3p	-0.99	-0.40	-0.32	1.94^−06^	*Efr3a*	1.28	0.01	1.40	0.00	0.49	0.01	1.11	4.17^−07^
	-0.99	-0.40	-0.32	1.94^−06^	*Serinc2*	1.16	1.52^−07^	1.47	0.01	1.03	7.30^−04^	1.43	4.02^−06^
	-0.99	-0.40	-0.32	1.94^−06^	*Tmem176b*	0.75	0.48	0.67	0.05	0.97	0.01	1.12	0.02
	-0.99	-0.40	-0.32	1.94^−06^	*Arl11*	0.86	0.01	0.08	0.75	0.72	0.01	0.71	0.07
	-0.99	-0.40	-0.32	1.94^−06^	*Pycard*	0.87	0.01	0.47	0.13	0.47	0.09	0.84	0.01
	-0.99	-0.40	-0.32	1.94^−06^	*Dhrs4*	0.20	0.10	0.11	0.57	0.28	0.03	-0.03	0.87
	-0.99	-0.40	-0.32	1.94^−06^	*Cd44*	0.11	0.09	0.01	0.99	0.68	0.02	0.18	0.67
	-0.99	-0.40	-0.32	1.94^−06^	*Anks1a*	0.72	0.03	0.53	0.05	0.28	0.19	0.17	0.53
	-0.99	-0.40	-0.32	1.94^−06^	*Asph*	0.45	0.08	0.45	0.03	0.17	0.13	0.05	0.90
	-0.99	-0.40	-0.32	1.94^−06^	*Cald1*	0.30	0.08	0.38	0.09	0.15	0.33	0.07	0.68
miR-138-5p	-0.48	-0.29	-0.50	3.98^−08^	*Rab32*	0.74	0.01	0.66	0.01	0.28	0.03	0.48	0.06
	-0.48	-0.29	-0.50	3.98^−08^	*Tpbg*	0.83	0.11	1.71	0.01	0.39	0.01	1.70	0.00
	-0.48	-0.29	-0.50	3.98^−08^	*Map3k14*	0.11	0.08	0.77	0.03	0.26	0.07	0.20	0.87
	-0.48	-0.29	-0.50	3.98^−08^	*Tcirg1*	0.05	0.82	0.36	0.07	0.46	0.06	-0.10	0.89
	-0.48	-0.29	-0.50	3.98^−08^	*Kank2*	0.62	0.02	0.71	0.07	0.59	0.02	2.91^−03^	0.95
	-0.48	-0.29	-0.50	3.98^−08^	*Ezh2*	0.16	0.27	0.40	0.07	0.31	0.05	0.04	0.78
	-0.48	-0.29	-0.50	3.98^−08^	*Spsb1*	-0.08	0.22	0.41	0.09	0.40	0.03	0.05	0.99
	-0.48	-0.29	-0.50	3.98^−08^	*C1qc*	1.11	1.37^−03^	0.42	0.34	0.97	0.08	0.92	0.18
	-0.48	-0.29	-0.50	3.98^−08^	*Sh3bgrl3*	0.77	0.01	0.49	0.13	0.48	0.00	0.70	0.01
	-0.48	-0.29	-0.50	3.98^−08^	*Zmiz1*	0.48	2.39^−03^	0.33	0.14	0.41	0.03	0.49	0.03
	-0.48	-0.29	-0.50	3.98^−08^	*Ly86*	0.87	0.03	0.40	0.41	0.97	0.03	0.58	0.05
	-0.48	-0.29	-0.50	3.98^−08^	*Fancd2os*	0.66	0.04	0.36	0.18	0.28	0.04	0.85	4.31^−03^
	-0.48	-0.29	-0.50	3.98^−08^	*Slc20a1*	0.41	0.02	0.14	0.52	0.45	0.00	0.27	0.09
	-0.48	-0.29	-0.50	3.98^−08^	*Rassf5*	0.36	0.08	0.00	1.00	0.32	0.05	0.46	0.43
	-0.48	-0.29	-0.50	3.98^−08^	*Cd44*	0.11	0.09	0.01	0.99	0.68	0.02	0.18	0.67
	-0.48	-0.29	-0.50	3.98^−08^	*Zfp521*	1.23	1.92^−04^	0.74	0.01	0.23	0.22	0.59	3.07^−03^
	-0.48	-0.29	-0.50	3.98^−08^	*Anks1a*	0.72	0.03	0.53	0.05	0.28	0.19	0.17	0.53
	-0.48	-0.29	-0.50	3.98^−08^	*Nnat*	1.09	0.01	0.86	0.07	0.15	0.59	0.63	0.30
	-0.48	-0.29	-0.50	3.98^−08^	*Htatip2*	0.70	0.01	0.37	0.07	-0.02	0.96	0.79	7.35^−04^
	-0.48	-0.29	-0.50	3.98^−08^	*Tnfsf9*	0.58	0.03	0.78	0.03	0.06	0.63	0.97	1.99^−04^
	-0.48	-0.29	-0.50	3.98^−08^	*Nabp1*	0.56	0.01	0.81	0.01	0.45	0.11	0.96	0.10
	-0.48	-0.29	-0.50	3.98^−08^	*Numbl*	0.37	0.06	0.40	0.03	0.20	0.14	0.43	0.32
	-0.48	-0.29	-0.50	3.98^−08^	*Cald1*	0.30	0.08	0.38	0.09	0.15	0.33	0.07	0.68
	-0.48	-0.29	-0.50	3.98^−08^	*Arpp21*	0.11	0.07	0.45	0.09	-0.02	0.91	0.63	0.02
miR-667-3p	-0.34	-0.20	-0.49	1.62^−07^	*Mdm1*	0.69	0.04	0.76	2.69^−03^	0.30	0.03	0.29	0.07
	-0.34	-0.20	-0.49	1.62^−07^	*Inpp4b*	0.80	0.10	1.15	2.69^−03^	0.31	0.07	0.24	0.13
	-0.34	-0.20	-0.49	1.62^−07^	*Tjp2*	0.40	0.15	0.78	0.02	0.42	0.05	0.19	0.56
	-0.34	-0.20	-0.49	1.62^−07^	*Serping1*	1.19	3.39^−03^	1.22	0.02	2.12	0.00	1.15	0.15
	-0.34	-0.20	-0.49	1.62^−07^	*Tmem176b*	0.75	0.48	0.67	0.05	0.97	0.01	1.12	0.02
	-0.34	-0.20	-0.49	1.62^−07^	*LOC500956*	0.34	0.20	0.50	0.07	0.27	0.02	0.12	0.91
	-0.34	-0.20	-0.49	1.62^−07^	*Chi3l1*	1.14	0.09	0.02	0.97	0.31	0.08	0.33	0.82
	-0.34	-0.20	-0.49	1.62^−07^	*Laptm5*	0.69	0.01	0.31	0.43	1.22	0.01	0.71	0.02
	-0.34	-0.20	-0.49	1.62^−07^	*Ifi30*	0.62	0.10	0.11	0.73	0.67	0.07	0.26	0.65
	-0.34	-0.20	-0.49	1.62^−07^	*Fcgr1a*	0.33	0.04	0.33	0.26	0.61	0.03	0.33	0.57
	-0.34	-0.20	-0.49	1.62^−07^	*Tmem140*	0.80	0.06	0.61	0.05	0.07	0.64	-0.25	0.92
miR-212-5p	2.55	0.27	0.28	1.15^−05^	*Rasd2*	-0.59	1.03^−03^	-1.24	1.19^−03^	-0.87	0.00	-0.58	3.80^−03^
	2.55	0.27	0.28	1.15^−05^	*Gabrd*	-0.53	0.01	-0.59	0.01	-0.55	0.01	-0.71	0.03
	2.55	0.27	0.28	1.15^−05^	*Hpca*	-0.74	0.02	-0.64	0.01	-0.22	0.02	-0.40	0.09

	2.55	0.27	0.28	1.15^−05^	*C1ql3*	0.14	0.57	-0.47	0.05	-0.38	0.00	-0.61	0.38
	2.55	0.27	0.28	1.15^−05^	*Fat1*	-1.05	1.24^−03^	-1.01	0.07	-0.19	0.08	-1.55	1.24^−03^
let-7b-3p	-0.80	-0.41	-5.75	8.36^−06^	*C1r*	0.87	8.05^−04^	0.79	0.02	0.81	0.01	0.54	0.01
	-0.80	-0.41	-5.75	8.36^−06^	*Gcnt1*	1.45	3.63^−05^	1.61	0.06	0.48	0.01	1.87	4.39^−04^
	-0.80	-0.41	-5.75	8.36^−06^	*Bdnf*	0.45	0.04	0.20	0.69	0.32	0.05	1.17	0.03
	-0.80	-0.41	-5.75	8.36^−06^	*Gpr83*	0.59	0.01	0.90	0.06	0.23	0.40	0.43	0.51
	-0.80	-0.41	-5.75	8.36^−06^	*Jup*	0.38	0.02	0.45	0.09	0.23	0.29	0.74	1.73^−03^
miR-132-3p	1.91	0.80	0.10	4.19^−18^	*Insig2*	-0.37	0.52	-0.81	0.01	-0.34	0.10	-0.15	0.80
miR-146a-5p	4.71	0.37	0.33	4.05^−10^	*Mthfd1l*	-0.94	5.30^−04^	-0.83	0.01	-0.44	0.01	-1.27	0.04
	4.71	0.37	0.33	4.05^−10^	*Plxdc1*	-0.28	0.06	-0.73	0.01	-0.33	0.08	-0.70	7.35^−04^
	4.71	0.37	0.33	4.05^−10^	*Htr5b*	-0.54	0.01	-1.66	0.03	-0.73	0.00	-1.55	3.87^−05^
	4.71	0.37	0.33	4.05^−10^	*B3galt5*	-1.39	7.49^−05^	-0.83	0.04	-0.43	0.05	-1.93	0.01
	4.71	0.37	0.33	4.05^−10^	*Pip5k1b*	-0.79	0.17	-0.46	0.05	-0.38	0.06	-1.03	0.02
miR-551b-3p	-0.78	-0.85	-0.42	9.09^−14^	*Efr3a*	1.28	0.01	1.40	5.22^−04^	0.49	0.01	1.11	4.17^−07^
	-0.78	-0.85	-0.42	9.09^−14^	*Rprm*	2.68	3.86^−05^	2.54	8.57^−04^	1.00	0.01	1.53	5.05^−04^
	-0.78	-0.85	-0.42	9.09^−14^	*Lox*	3.11	2.13^−09^	2.56	2.69^−03^	1.28	0.00	2.66	6.51^−06^
	-0.78	-0.85	-0.42	9.09^−14^	*Sox11*	1.57	0.01	1.36	0.01	1.23	0.00	1.52	2.72^−03^
	-0.78	-0.85	-0.42	9.09^−14^	*Tpbg*	0.83	0.11	1.71	0.01	0.39	0.01	1.70	3.06^−03^
	-0.78	-0.85	-0.42	9.09^−14^	*C1qc*	1.11	1.37^−03^	0.42	0.34	0.97	0.08	0.92	0.18
	-0.78	-0.85	-0.42	9.09^−14^	*Syn2*	0.65	0.04	0.55	0.03	0.11	0.21	0.39	0.03
	-0.78	-0.85	-0.42	9.09^−14^	*Nabp1*	0.56	0.01	0.81	0.01	0.45	0.11	0.96	0.10
	-0.78	-0.85	-0.42	9.09^−14^	*Ntm*	0.71	0.05	0.37	0.09	-0.22	0.37	-0.32	0.94
miR-3573-3p	-1.02	-0.26	-0.93	4.68^−17^	*Serping1*	1.19	3.39^−03^	1.22	0.02	2.12	0.00	1.15	0.15
	-1.02	-0.26	-0.93	4.68^−17^	*Map3k4*	0.09	0.59	0.53	0.03	0.29	0.07	0.02	1.00
	-1.02	-0.26	-0.93	4.68^−17^	*Col6a3*	0.31	0.01	0.79	0.08	0.35	0.06	0.63	0.09
	-1.02	-0.26	-0.93	4.68^−17^	*Plcxd3*	1.94	3.86^−05^	1.58	0.17	0.59	0.01	2.02	0.01
	-1.02	-0.26	-0.93	4.68^−17^	*S100a10*	1.36	1.68^−03^	0.61	0.50	0.80	0.02	1.09	0.43
	-1.02	-0.26	-0.93	4.68^−17^	*Chi3l1*	1.14	0.09	0.02	0.97	0.31	0.08	0.33	0.82
	-1.02	-0.26	-0.93	4.68^−17^	*Rbms1*	1.24	1.76^−03^	0.65	0.17	0.50	0.08	0.59	0.34
	-1.02	-0.26	-0.93	4.68^−17^	*Lgmn*	0.41	0.03	0.09	0.77	0.61	0.06	0.22	0.62
	-1.02	-0.26	-0.93	4.68^−17^	*P2ry6*	0.17	0.05	0.12	0.72	0.61	0.02	0.50	0.16
	-1.02	-0.26	-0.93	4.68^−17^	*Epb41l4b*	0.70	8.37^−04^	0.58	0.02	0.17	0.46	0.57	2.98^−03^
miR-139-5p	-1.40	-0.77	-0.91	4.40^−17^	*C1s*	1.55	1.42^−06^	1.15	2.00^−03^	0.55	0.02	1.34	3.94^−05^
	-1.40	-0.77	-0.91	4.40^−17^	*Tmem176b*	0.75	0.48	0.67	0.05	0.97	0.01	1.12	0.02
	-1.40	-0.77	-0.91	4.40^−17^	*Slc7a14*	0.91	3.46^−04^	0.42	0.09	0.23	0.06	0.35	0.17
	-1.40	-0.77	-0.91	4.40^−17^	*Fcgr2b*	1.87	3.62^−04^	0.99	0.20	1.52	0.01	1.39	0.01
	-1.40	-0.77	-0.91	4.40^−17^	*C5ar1*	0.44	0.02	0.33	0.31	0.24	0.08	0.43	0.28
	-1.40	-0.77	-0.91	4.40^−17^	*Syn2*	0.65	0.04	0.55	0.03	0.11	0.21	0.39	0.03
	-1.40	-0.77	-0.91	4.40^−17^	*Anks1a*	0.72	0.03	0.53	0.05	0.28	0.19	0.17	0.53
	-1.40	-0.77	-0.91	4.40^−17^	*Mtmr11*	0.95	3.62^−04^	1.86	0.01	0.05	0.87	1.13	4.82^−05^
miR-33-5p	-2.40	-0.80	-0.51	4.92^−19^	*Runx1*	1.08	7.92^−04^	1.48	2.69^−03^	0.60	2.69^−03^	1.22	8.95^−04^
	-2.40	-0.80	-0.51	4.92^−19^	*Wnt10a*	0.58	1.89^−03^	0.81	0.07	0.46	0.01	1.69	0.01
	-2.40	-0.80	-0.51	4.92^−19^	*Slc7a14*	0.91	3.46^−04^	0.42	0.09	0.23	0.06	0.35	0.17
	-2.40	-0.80	-0.51	4.92^−19^	*Fcgr2b*	1.87	3.62^−04^	0.99	0.20	1.52	0.01	1.39	0.01
	-2.40	-0.80	-0.51	4.92^−19^	*Ly86*	0.87	0.03	0.40	0.41	0.97	0.03	0.58	0.05
	-2.40	-0.80	-0.51	4.92^−19^	*Cfh*	1.14	0.04	0.91	0.21	0.70	0.03	0.70	0.11
miR-7a-5p	-0.91	-0.93	-0.51	3.44^−24^	*Mdm1*	0.69	0.04	0.76	2.69^−03^	0.30	0.03	0.29	0.07
	-0.91	-0.93	-0.51	3.44^−24^	*Wls*	1.45	7.49^−05^	0.80	0.01	0.57	0.01	1.75	1.52^−05^
	-0.91	-0.93	-0.51	3.44^−24^	*Tpbg*	0.83	0.11	1.71	0.01	0.39	0.01	1.70	3.06^−03^
	-0.91	-0.93	-0.51	3.44^−24^	*Serping1*	1.19	3.39^−03^	1.22	0.02	2.12	0.00	1.15	0.15
	-0.91	-0.93	-0.51	3.44^−24^	*Map3k14*	0.11	0.08	0.77	0.03	0.26	0.07	0.20	0.87
	-0.91	-0.93	-0.51	3.44^−24^	*Tmem176b*	0.75	0.48	0.67	0.05	0.97	0.01	1.12	0.02
	-0.91	-0.93	-0.51	3.44^−24^	*Dnah12*	0.32	0.41	0.65	0.05	0.22	0.03	-0.35	0.91
	-0.91	-0.93	-0.51	3.44^−24^	*Pafah1b3*	0.88	0.04	0.84	0.09	0.28	0.01	0.42	0.79
	-0.91	-0.93	-0.51	3.44^−24^	*S100a4*	1.44	2.54^−07^	0.53	0.17	0.67	0.01	1.05	0.09
	-0.91	-0.93	-0.51	3.44^−24^	*C1qa*	1.21	0.05	0.34	0.29	1.27	0.04	1.16	3.03^−03^
	-0.91	-0.93	-0.51	3.44^−24^	*Gpnmb*	1.54	3.04^−03^	0.30	0.68	1.45	0.03	0.81	0.22
	-0.91	-0.93	-0.51	3.44^−24^	*Resp18*	0.65	1.68^−03^	0.38	0.23	0.28	0.09	0.82	0.02
	-0.91	-0.93	-0.51	3.44^−24^	*Cfh*	1.14	0.04	0.91	0.21	0.70	0.03	0.70	0.11
	-0.91	-0.93	-0.51	3.44^−24^	*Arhgdib*	0.27	0.01	0.48	0.34	0.85	0.03	0.49	0.19
	-0.91	-0.93	-0.51	3.44^−24^	*Ick*	0.17	0.04	0.28	0.12	0.27	0.10	-0.01	0.88
	-0.91	-0.93	-0.51	3.44^−24^	*Nubpl*	1.06	1.77^−04^	0.88	2.44^−03^	0.06	0.64	0.77	0.02
	-0.91	-0.93	-0.51	3.44^−24^	*Arpp21*	0.11	0.07	0.45	0.09	-0.02	0.91	0.63	0.02
miR-212-3p	1.09	0.88	0.22	1.74^−47^	*Insig2*	-0.37	0.52	-0.81	0.01	-0.34	0.10	-0.15	0.80

The relationship between the changes in expression of miRNAs and their mRNA targets in the chronic stage of epilepsy was analyzed using only the amygdala stimulation dataset (Bot et al., 2013). We observed negative correlations (based on fold changes) between all five miRNAs that emerged as significantly downregulated from the meta-analysis and 29 unique predicted mRNA targets in the dataset. Five of these 29 anticorrelated mRNAs were predicted targets and had inverse expression relative to two miRNAs and one, the glutamate ionotropic receptor δ-type subunit 2 (*Grid2*), was a predicted target and had inverse expression relative to three miRNAs, namely, miR-130a-3p, miR-148b-3p, and miR-551b-3p (Table 5). Furthermore, interestingly, three mRNA targets, the transmembrane protein 176B (*Tmem176b*), the EFR3 homolog A (*Efr3a*), and the zinc finger, MIZ-type containing 1 (*Zmiz1*) were downregulated in both epileptogenesis and the chronic stage.

**Table 5.** miRNA-mRNA fold changes inverse correlation at the chronic stage

miRNA name	Pilocarpine	Angular bundle stimulation	Amygdala stimulation		mRNA gene name	Amygdala Stimulation	
	miRNA FC			Meta-analysis adjusted *p* value		FC	adjusted *p* value
miR-130a-3p	-0.24	-0.32	-0.46	0.0107	*Frmd6*	0.35	0.0077
	-0.24	-0.32	-0.46	0.0107	*Grid2*	0.31	0.0702
	-0.24	-0.32	-0.46	0.0107	*Necab3*	0.43	0.0202
	-0.24	-0.32	-0.46	0.0107	*Npepl1*	0.22	0.0847
miR-148b-3p	-0.24	-0.29	-0.41	0.0317	*C1qa*	0.81	0.0559
	-0.24	-0.29	-0.41	0.0317	*Ctsz*	0.63	0.0773
	-0.24	-0.29	-0.41	0.0317	*Flnc*	0.53	0.0455
	-0.24	-0.29	-0.41	0.0317	*Frmd6*	0.35	0.0077
	-0.24	-0.29	-0.41	0.0317	*Grid2*	0.31	0.0702
	-0.24	-0.29	-0.41	0.0317	*Npepl1*	0.22	0.0847
	-0.24	-0.29	-0.41	0.0317	*Tax1bp3*	0.36	0.0371
	-0.24	-0.29	-0.41	0.0317	*Tmem176b*	0.91	0.0621
miR-324-3p	-0.44	-0.22	-0.27	0.0055	*Acss1*	0.31	0.0899
	-0.44	-0.22	-0.27	0.0055	*Atraid*	0.23	0.0936
	-0.44	-0.22	-0.27	0.0055	*Cd9*	0.46	0.0380
	-0.44	-0.22	-0.27	0.0055	*Chi3l1*	0.45	0.0455
	-0.44	-0.22	-0.27	0.0055	*Csf1r*	0.80	0.0773
	-0.44	-0.22	-0.27	0.0055	*Ctsb*	0.20	0.0918
	-0.44	-0.22	-0.27	0.0055	*Gfap*	0.93	0.0380
	-0.44	-0.22	-0.27	0.0055	*Gsap*	0.20	0.0843
	-0.44	-0.22	-0.27	0.0055	*Hmox1*	0.19	0.0972
	-0.44	-0.22	-0.27	0.0055	*Limd2*	0.26	0.0817
	-0.44	-0.22	-0.27	0.0055	*Mex3b*	0.33	0.0217
	-0.44	-0.22	-0.27	0.0055	*Osbpl9*	0.19	0.0760
	-0.44	-0.22	-0.27	0.0055	*Slco2b1*	0.46	0.0896
	-0.44	-0.22	-0.27	0.0055	*Tmem176b*	0.91	0.0621
	-0.44	-0.22	-0.27	0.0055	*Zmiz1*	0.34	0.0077
miR-551b-3p	-1.30	-0.52	-0.50	0.0006	*Csf1r*	0.80	0.0773
	-1.30	-0.52	-0.50	0.0006	*Efr3a*	0.60	0.0027
	-1.30	-0.52	-0.50	0.0006	*Entpd2*	0.44	0.0518
	-1.30	-0.52	-0.50	0.0006	*Grid2*	0.31	0.0702
	-1.30	-0.52	-0.50	0.0006	*Npc2*	0.80	0.0882
	-1.30	-0.52	-0.50	0.0006	*Sox11*	0.86	0.0455
miR-652-3p	-0.90	-0.34	-0.41	9.65^−07^	*Cd9*	0.46	0.0380
	-0.90	-0.34	-0.41	9.65^−07^	*Hsd3b7*	0.31	0.0402
	-0.90	-0.34	-0.41	9.65^−07^	*Tmem176a*	0.93	0.0559

Recent evidence supports the notion that miRNAs not only decrease levels of their mRNA targets (Guo et al., 2010), but additionally may have nuclear functions capable of influencing gene expression, and which may be reflected by a correlation between a miRNA and its target gene mRNA levels (Catalanotto et al., 2016). Analysis of the epileptogenesis data revealed significant correlation (gene FDR < 0.1), for 21 (of 26) miRNAs and 77 unique predicted gene targets in at least three of the four epilepsy models of the Dingledine dataset (Dingledine et al., 2017; Table 6). In addition, we found positive correlations between five of the five miRNAs that were downregulated in the chronic period and 39 predicted mRNA targets in the amygdala stimulation dataset (Bot et al., 2013; Table 7). Interestingly, 29 of the mRNAs identified as potential targets in epileptogenesis were inversely correlated to some miRNAs and directly correlated to others (e.g., *map3k14* is inversely correlated to miR-7a-5p and miR-138-5p and directly correlated to miR-212-5p, while *bdnf* is inversely correlated to let-7b-3p and directly correlated to miR-212-5p). This observation prompts the hypothesis that some mRNAs may be subject to a dual control by different miRNAs at cytosolic and nuclear level. This hypothesis should be challenged and investigated.

**Table 6.** miRNA-mRNA fold changes positive correlation in epileptogenesis

miRNA Name	microRNA data				mRNA targets of respective microRNA (FDR < 0.1)								
	Pilocarpine	Perforant path stimulation	Amygdala stimulation		mRNA gene names	Pilocarpine		Perforant path stimulation		Amygdala stimulation		Kainic acid	
		miRNA FC		Meta-analysis *p* value		FC	Adjusted *p* value	FC	Adjusted *p* value	FC	Adjusted *p* value	FC	Adjusted *p* value
miR-383-5p	-0.50	-0.35	-0.75	6.11^−05^	*Rasd2*	-0.58	0.0038	-1.24	0.0012	-0.59	0.0010	-0.87	0.0034
					*Sec14l1*	-0.59	0.0061	-0.50	0.0425	-0.39	0.0053	-0.20	0.0726
					*Mpp6*	-0.59	0.0295	-0.54	0.0517	-0.39	0.4123	-0.78	0.0510
miR-153-3p	-0.32	-0.42	-0.22	0.0008	*Mthfd1l*	-1.27	0.0398	-0.83	0.0102	-0.94	0.0005	-0.44	0.0093
					*Gdf10*	-2.91	8.62^−06^	-1.46	0.0306	-1.86	2.27^−07^	-1.02	0.0014
					*Nr4a3*	0.12	0.9726	-1.65	0.0721	-1.64	0.0574	-0.65	0.0042
					*Mettl7a*	-0.74	0.0066	-0.72	0.0861	-0.87	0.0750	-0.19	0.0500
					*Ablim2*	-0.05	0.9953	-0.78	0.0964	-0.35	0.7323	-0.29	0.0667
miR-324-5p	-0.09	-0.60	-0.29	8.552^−06^	*Ryr1*	-1.52	0.0022	-1.39	0.0196	-1.86	2.53^−08^	-0.68	0.0014
miR-150-5p	-0.45	-0.45	-0.56	6.168^−05^	*Ddit4l*	-2.38	0.0063	-1.89	0.0098	-2.00	6.09^−06^	-1.09	0.0007
					*Htr5b*	-1.55	3.87^−05^	-1.66	0.0263	-0.54	0.0096	-0.73	0.0016
					*Fkbp4*	-0.21	0.7718	-0.55	0.0464	-0.45	0.0737	-0.26	0.0775
					*Pip5k1b*	-1.03	0.0228	-0.46	0.0492	-0.79	0.1731	-0.38	0.0619
					*Calml4*	-0.60	0.3155	-0.44	0.0839	-0.48	0.1135	-0.38	0.0139
miR-92b-3p	-1.05	-0.72	-0.37	1.695^−05^	*Per2*	-0.59	0.8691	-0.46	0.0717	-0.56	0.0253	-0.37	0.0918
miR-345-5p	-0.23	-0.25	-0.12	1.464^−06^	*Rasd2*	-0.58	0.0038	-1.24	0.0012	-0.59	0.0010	-0.87	0.0034
					*Ryr1*	-1.52	0.0022	-1.39	0.0196	-1.86	2.53^−08^	-0.68	0.0014
					*Klhl14*	-2.36	2.39^−06^	-1.66	0.0204	-0.84	0.0006	-0.44	0.0077
					*B3galt5*	-1.93	0.0118	-0.83	0.0442	-1.39	7.49^−05^	-0.43	0.0471
					*Pip5k1b*	-1.03	0.0228	-0.46	0.0492	-0.79	0.1731	-0.38	0.0619
					*Rspo3*	-0.41	0.5671	-0.41	0.0626	-0.11	0.1711	-0.55	0.0259
					*Fat1*	-1.55	0.0012	-1.01	0.0717	-1.05	0.0012	-0.19	0.0792
miR-101a-3p	-0.68	-0.71	-0.19	5.148^−06^	*Rasd2*	-0.58	0.0038	-1.24	0.0012	-0.59	0.0010	-0.87	0.0034
					*Ddit4l*	-2.38	0.0063	-1.89	0.0098	-2.00	6.09^−06^	-1.09	0.0007
					*Gdf10*	-2.91	8.62^−06^	-1.46	0.0306	-1.86	2.27^−07^	-1.02	0.0014
					*Plk5*	-2.15	0.0033	-0.59	0.0311	-1.46	0.0013	-1.00	0.0303
					*Plag1*	-0.58	0.0528	-0.45	0.0425	0.06	0.5075	-0.59	0.0453
miR-29c-5p	-0.59	-0.41	-0.14	1.477^−07^	*Crim1*	-1.07	0.0084	-0.59	0.0173	-0.87	9.88^−06^	-0.31	0.0323
					*Dnah12*	-0.35	0.9054	0.65	0.0492	0.32	0.4073	0.22	0.0340
					*C5ar1*	0.43	0.2817	0.33	0.3103	0.44	0.0181	0.24	0.0841
					*Slc20a1*	0.27	0.0883	0.14	0.5191	0.41	0.0227	0.45	0.0042
miR-330-3p	-0.99	-0.40	-0.32	1.935^−06^	*Gabrd*	-0.71	0.0282	-0.59	0.0067	-0.53	0.0053	-0.55	0.0070
					*Ets2*	-0.44	0.3991	-0.66	0.0249	-0.44	0.0641	-0.31	0.0095
					*Gpc3*	-1.92	0.0026	-0.85	0.0492	-0.89	0.0006	-1.29	0.0054
miR-138-5p	-0.48	-0.29	-0.50	3.983^−08^	*Rasd2*	-0.58	0.0038	-1.24	0.0012	-0.59	0.0010	-0.87	0.0034
					*Nr4a1*	0.24	0.7205	-0.88	0.0125	-0.56	0.4123	-0.32	0.0719
					*Crim1*	-1.07	0.0084	-0.59	0.0173	-0.87	9.88^−06^	-0.31	0.0323
					*Nhlh1*	-1.67	0.0023	-1.12	0.0337	-0.63	0.0295	-0.84	0.0097
					*Nr4a3*	0.12	0.9726	-1.65	0.0721	-1.64	0.0574	-0.65	0.0042
miR-667-3p	-0.34	-0.20	-0.49	1.62^−07^	*Rasd2*	-0.58	0.0038	-1.24	0.0012	-0.59	0.0010	-0.87	0.0034
					*Etv5*	-0.08	0.9646	-0.63	0.0423	-0.47	0.2965	-0.49	0.0044
miR-212-5p	2.55	0.27	0.28	1.152^−05^	*Sox11*	1.52	0.0027	1.36	0.0092	1.57	0.0063	1.23	0.0011
					*Serping1*	1.15	0.1500	1.22	0.0204	1.19	0.0034	2.12	0.0009
					*Map3k14*	0.20	0.8682	0.77	0.0263	0.11	0.0834	0.26	0.0653
					*Ptprn*	0.83	0.0001	0.56	0.0613	0.38	0.0034	0.33	0.0648
					*Kank2*	0.00	0.9473	0.71	0.0673	0.62	0.0153	0.59	0.0241
					*Acan*	0.30	0.0723	0.37	0.0799	0.17	0.2577	0.37	0.0436
					*Slc7a14*	0.35	0.1717	0.42	0.0877	0.91	0.0003	0.23	0.0568
					*C1qc*	0.92	0.1759	0.42	0.3397	1.11	0.0014	0.97	0.0816
					*Ly86*	0.58	0.0494	0.40	0.4124	0.87	0.0299	0.97	0.0299
					*Slc20a1*	0.27	0.0883	0.14	0.5191	0.41	0.0227	0.45	0.0042
					*Blnk*	0.70	0.0039	0.43	0.1925	0.71	0.0377	0.78	0.0158
					*Bdnf*	1.17	0.0297	0.20	0.6933	0.45	0.0352	0.32	0.0487
					*Pdlim4*	0.19	0.7034	0.15	0.5799	0.15	0.0771	0.46	0.0721
					*Syn2*	0.39	0.0258	0.55	0.0299	0.65	0.0399	0.11	0.2134
					*Epb41l4b*	0.57	0.0030	0.58	0.0237	0.70	0.0008	0.17	0.4555
					*Nnat*	0.63	0.2970	0.86	0.0717	1.09	0.0083	0.15	0.5894
					*Htatip2*	0.79	0.0007	0.37	0.0669	0.70	0.0119	-0.02	0.9557
					*Trh*	2.87	0.0096	1.31	0.0246	1.20	0.0145	0.99	0.3361
					*Asph*	0.05	0.9026	0.45	0.0254	0.45	0.0832	0.17	0.1308
let-7b-3p	-0.80	-0.41	-5.75	8.364^−06^	*Rspo3*	-0.41	0.5671	-0.41	0.0626	-0.11	0.1711	-0.55	0.0259

miR-132-3p	1.91	0.80	0.10	4.195^−18^	*Efr3a*	1.11	4.17^−07^	1.40	0.0005	1.28	0.0096	0.49	0.0070
					*Sox11*	1.52	0.0027	1.36	0.0092	1.57	0.0063	1.23	0.0011
					*Wls*	1.75	1.52^−05^	0.80	0.0108	1.45	7.49^−05^	0.57	0.0051
					*Rin2*	-0.06	0.9546	0.43	0.0517	0.57	0.0638	0.44	0.0303
					*Gpnmb*	0.81	0.2150	0.30	0.6840	1.54	0.0030	1.45	0.0259
					*Zfp521*	0.59	0.0031	0.74	0.0052	1.23	0.0002	0.23	0.2169
					*Asph*	0.05	0.9026	0.45	0.0254	0.45	0.0832	0.17	0.1308
miR-146a-5p	4.71	0.37	0.33	4.047^−10^	*Mdm1*	0.29	0.0680	0.76	0.0027	0.69	0.0360	0.30	0.0322
					*Inpp4b*	0.24	0.1291	1.15	0.0027	0.80	0.1003	0.31	0.0735
					*Arhgap17*	0.33	0.0762	0.88	0.0080	0.30	0.0183	0.43	0.0259
					*Igsf1*	0.50	0.4934	0.89	0.0425	1.03	0.3719	0.31	0.0510
					*Gpat3*	0.44	0.4497	0.40	0.0864	-0.04	0.2317	0.16	0.0923
					*Sowahc*	0.53	0.0683	0.45	0.3070	1.09	0.0006	0.28	0.0873
					*Fcer1g*	1.09	0.0112	0.51	0.1380	1.14	0.0002	1.37	0.0059
					*Slfn13*	0.40	0.1166	0.41	0.1757	0.46	0.0429	1.53	0.0027
					*Zfp521*	0.59	0.0031	0.74	0.0052	1.23	0.0002	0.23	0.2169
					*Anks1a*	0.17	0.5280	0.53	0.0492	0.72	0.0323	0.28	0.1948
					*Trh*	2.87	0.0096	1.31	0.0246	1.20	0.0145	0.99	0.3361
					*Ntm*	-0.32	0.9428	0.37	0.0861	0.71	0.0527	-0.22	0.3746
					*Col9a1*	1.28	0.0003	0.91	0.0425	0.41	0.0208	0.03	0.9192
miR-551b-3p	-0.78	-0.85	-0.42	9.092^−14^	*Plxdc1*	-0.70	0.0007	-0.73	0.0127	-0.28	0.0555	-0.33	0.0823
					*Ogfrl1*	-0.23	0.1736	-0.52	0.0135	-0.55	0.0293	-0.30	0.0563
					*Htr5b*	-1.55	3.87^−05^	-1.66	0.0263	-0.54	0.0096	-0.73	0.0016
					*Diaph1*	-0.27	0.2293	-0.30	0.0492	0.07	0.3960	-0.23	0.0530
miR-344b-2-3p	3.74	0.11	0.29	5.432^−14^	*Runx1*	1.22	0.0009	1.48	0.0027	1.08	0.0008	0.60	0.0027
					*Il18*	0.31	0.3608	0.52	0.0669	0.38	0.1009	0.51	0.0955
					*Chi3l1*	0.33	0.8228	0.02	0.9731	1.14	0.0881	0.31	0.0785
					*Fancd2os*	0.85	0.0043	0.36	0.1786	0.66	0.0353	0.28	0.0372
					*P2ry6*	0.50	0.1614	0.12	0.7169	0.17	0.0499	0.61	0.0231
					*Asph*	0.05	0.9026	0.45	0.0254	0.45	0.0832	0.17	0.1308
miR-139-5p	-1.40	-0.77	-0.91	4.4^−17^	*Gabrd*	-0.71	0.0282	-0.59	0.0067	-0.53	0.0053	-0.55	0.0070
					*Gdf10*	-2.91	8.62^−06^	-1.46	0.0306	-1.86	2.27^−07^	-1.02	0.0014
					*Rspo3*	-0.41	0.5671	-0.41	0.0626	-0.11	0.1711	-0.55	0.0259
miR-33-5p	-2.40	-0.80	-0.51	4.916^−19^	*Smarca2*	-0.60	0.2192	-1.53	0.0186	-0.35	0.0904	-0.27	0.0340
					*Fxyd7*	-1.49	7.16^−05^	-1.65	0.0186	-0.98	0.0052	-0.30	0.0142
					*Arg1*	-1.24	0.0324	-0.49	0.0984	-0.37	0.0679	-0.50	0.0344
miR-7a-5p	-0.91	-0.93	-0.51	3.436^−24^	*Hpca*	-0.40	0.0880	-0.64	0.0090	-0.74	0.0220	-0.22	0.0244
miR-212-3p	1.09	0.88	0.22	1.744^−47^	*Efr3a*	1.11	4.17^−07^	1.40	0.0005	1.28	0.0096	0.49	0.0070
					*Sox11*	1.52	0.0027	1.36	0.0092	1.57	0.0063	1.23	0.0011
					*Wls*	1.75	1.52^−05^	0.80	0.0108	1.45	7.49^−05^	0.57	0.0051
					*Rin2*	-0.06	0.9546	0.43	0.0517	0.57	0.0638	0.44	0.0303
					*Gpnmb*	0.81	0.2150	0.30	0.6840	1.54	0.0030	1.45	0.0259
					*Zfp521*	0.59	0.0031	0.74	0.0052	1.23	0.0002	0.23	0.2169
					*Asph*	0.05	0.9026	0.45	0.0254	0.45	0.0832	0.17	0.1308

**Table 7.** miRNA-mRNA fold changes positive correlation in the chronic period

miRNA name	microRNA data				mRNA targets of respective microRNA (FDR < 0.1)		
	Pilocarpine	Perforant path stimulation	Amygdala stimulation		mRNA gene names	Amygdala stimulation	Amygdala stimulation
		FC		Meta-analysis *p* value		FC	Adjusted *p* value
miR-652-3p	-0.90	-0.34	-0.41	9.65^−07^	*Ano2*	-0.39	0.0077
					*Ece2*	-0.22	0.0395
					*Optn*	-0.26	0.0825
miR-551b-3p	-1.30	-0.52	-0.50	0.0006	*Clmp*	-0.36	0.0325
					*Socs5*	-0.36	0.0619
					*Asic2*	-0.26	0.0731
miR-324-3p	-0.44	-0.22	-0.27	0.0055	*Gdf10*	-0.94	0.0083
					*Gpr176*	-0.43	0.0116
					*Etv5*	-0.58	0.0202
					*Elfn2*	-0.25	0.0225
					*Tcerg1l*	-0.42	0.0311
					*Sstr3*	-0.33	0.0371
					*Nr4a3*	-0.50	0.0372
					*Veph1*	-0.27	0.0380
					*Cyp26b1*	-0.35	0.0380
					*Itgb4*	-0.50	0.0394
					*Nefm*	-0.31	0.0394
					*Alcam*	-0.29	0.0547
					*Grik1*	-0.30	0.0555
					*Clmn*	-0.30	0.0619
					*Arg1*	-0.30	0.0697
					*Grik3*	-0.72	0.0702
					*Asic2*	-0.26	0.0731
					*Boc*	-0.33	0.0772
					*Ubash3b*	-0.24	0.0773
					*Cbarp*	-0.18	0.0817
miR-148b-3p	-0.24	-0.29	-0.41	0.0317	*Pip5k1b*	-0.50	0.0077
					*Gpr176*	-0.43	0.0116
					*Slit2*	-0.39	0.0219
					*Camk1g*	-0.43	0.0234
					*Gpr165*	-0.48	0.0326
					*Hcrtr2*	-0.28	0.0371
					*Htra4*	-1.37	0.0380
					*Vstm2b*	-0.40	0.0504
					*Alcam*	-0.29	0.0547
					*Ankrd34c*	-0.44	0.0568
					*Ppara*	-0.25	0.0773
					*Hnrnpm*	-0.22	0.0988
miR-130a-3p	-0.24	-0.32	-0.46	0.0107	*Eloc*	-0.33	0.0380
					*Htra4*	-1.37	0.0380
					*Trhr*	-0.75	0.0380
					*Mthfd1l*	-0.39	0.0505
					*Rasd2*	-0.47	0.0560

### Genes that are anticorrelated with miRNAs are enriched for “epileptogenic” ontology categories

To further investigate the functional role of miRNAs significantly differentially expressed and anticorrelated with their predicted mRNA targets, we examined the functional enrichment of the mRNA targets identified in epileptogenesis and chronic phases of epilepsy.

**Figure 5.**
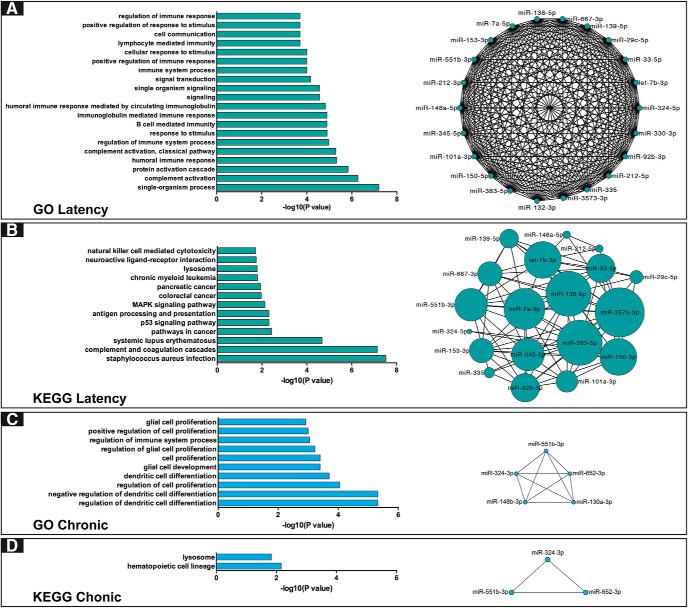
Functional enrichment of dysregulated miRNA-mRNAs targets modules. ***A***, Horizontal bar plots (on the left) show the GO enrichment status (top 20 terms) for 112 predicted mRNAs targets that anticorrelate with 22 miRNAs expression level in epileptogenesis (FDR < 5%, hypergeometric test). The miRNA-mRNA module is represented by a network graph (on the right) showing the connections between miRNAs based on the function of their mRNAs predictive targets revealed by the GO enrichment. ***B***, Horizontal bar plots (on the left) show KEGG enrichment analysis for predicted mRNAs targets that anticorrelate with miRNAs expression level in epileptogenesis (FDR < 5%, hypergeometric test). miRNA-mRNA modules are represented with network plot (on the right) showing the connection between miRNAs based on the pathways in which are involved their predicted targets revealed by KEGG analysis. ***C***, ***D***, GO and KEGG enrichment status for 29 predicted miRNA targets that anticorrelate with five miRNAs differentially expressed in the chronic stage (FDR < 5%, hypergeometric test).

Target genes that inversely correlated with differentially expressed miRNAs during the epileptogenesis period were enriched for GO terms related to synaptic function [like “response to stimulus” (*p* = 0.0013), “signaling” (*p* = 2.68^−05^), “signal transduction” (*p* = 0.0047), and others] and immunity [like “humoral immune response” (*p* = 0.0009), “regulation of immune system process” (*p* = 0.0013), and others]. In addition, terms related to complement activation [like “complement activation” (*p* = 0.0002) and “complement activation, classical pathway” (*p* = 0.0009)] are in prominent position (Fig. 5*A*; Table 8). Proteins of the classical complement pathway not only play a role in the innate immune system, but have been also shown to be released from neurons, and serve as a new class of synaptic organizers (Yuzaki, 2017). These GO terms are potentially relevant to changes occurring at the level of the DG in epileptogenesis (Dudek and Sutula, 2007; Vezzani et al., 2015). In addition, at the level of cell signaling pathways, analysis of KEGG pathways enriched among the mRNA targets suggested a key role for the MAPK cascade (*p* = 0.354; Fig. 5*B*; Table 8). Notably, changes in the activation state of kinase pathways and altered kinase expression patterns have been reported in the hippocampus by previous studies (Xi et al., 2009). In the chronic period, the predicted and anticorrelated mRNA targets revealed enrichment in biological processes that have been previously implicated in chronic epilepsy (Ludewig et al., 2016; Robel and Sontheimer, 2016) such as “regulation of dendritic cell differentiation” (*p* = 4.8 × 10^−6^), “glial cell development” (*p* = 4.0 × 10^−4^), “proliferation” (*p* = 6.0 × 10^−4^), and “cell proliferation” (*p* = 4.0 × 10^−4^; Fig. 5*C*; Table 9). Notably, we performed a permutation test to check for false positive enrichment in GO term and KEGG pathway analysis, but this did not change any of the results.

**Table 8.** GO and KEGG enrichment of 122 predicted and anticorrelated mRNAs targets of 22 miRNAs differentially expressed in epileptogenesis

GO terms	Term description	GO ID	Size of term	miRNA target	Expected	Enrichment ratio	Raw *p* value	FDR
Biological process	Single-organism process	GO:004469	C = 2489	O = 67	E = 42.21	R = 1.59	6.29^−08^	5.71^−05^
Biological process	Complement activation	GO:000695	C = 11	O = 5	E = 0.19	R = 26.80	5.34^−07^	0.0002
Biological process	Protein activation cascade	GO:007237	C = 13	O = 5	E = 0.22	R = 22.68	1.45^−06^	0.0004
Biological process	Humoral immune response	GO:000695	C = 16	O = 5	E = 0.27	R = 18.43	4.72^−06^	0.0009
Biological process	Complement activation, classical pathway	GO:000695	C = 8	O = 4	E = 0.14	R = 29.48	5.14^−06^	0.0009
Biological process	B cell-mediated immunity	GO:001972	C = 32	O = 6	E = 0.54	R = 11.06	1.28^−05^	0.0013
Biological process	Regulation of immune system process	GO:000268	C = 242	O = 15	E = 4.10	R = 3.65	1.05^−05^	0.0013
Biological process	Response to stimulus	GO:005089	C = 2294	O = 59	E = 38.90	R = 1.52	1.25^−05^	0.0013
Biological process	Immunoglobulin-mediated immune response	GO:001606	C = 32	O = 6	E = 0.54	R = 11.06	1.28^−05^	0.0013
Biological process	Humoral immune response mediated by circulating immunoglobulin	GO:000245	C = 10	O = 4	E = 0.17	R = 23.59	1.50^−05^	0.0014
Biological process	Signaling	GO:002305	C = 1510	O = 44	E = 25.61	R = 1.72	2.68^−05^	0.002
Biological process	Single-organism signaling	GO:004470	C = 1510	O = 44	E = 25.61	R = 1.72	2.68^−05^	0.002
Biological process	Signal transduction	GO:000716	C = 1308	O = 39	E = 22.18	R = 1.76	6.75^−05^	0.0047
Biological process	Immune system process	GO:000237	C = 446	O = 19	E = 7.56	R = 2.51	0.0001	0.0057
Biological process	Positive regulation of immune response	GO:005077	C = 90	O = 8	E = 1.53	R = 5.24	0.0001	0.0057
Biological process	Cellular response to stimulus	GO:005171	C = 1711	O = 46	E = 29.02	R = 1.59	0.0001	0.0057
Biological process	Lymphocyte mediated immunity	GO:000244	C = 51	O = 6	E = 0.86	R = 6.94	0.0002	0.0086
Biological process	Cell communication	GO:000715	C = 1570	O = 43	E = 26.63	R = 1.62	0.0002	0.0086
Biological process	Positive regulation of response to stimulus	GO:004858	C = 380	O = 17	E = 6.44	R = 2.64	0.0002	0.0086
Biological process	Regulation of immune response	GO:005077	C = 125	O = 9	E = 2.12	R = 4.25	0.0002	0.0086
Biological process	Immune effector process	GO:000225	C = 120	O = 9	E = 2.04	R = 4.42	0.0002	0.0086
Biological process	Immune response	GO:000695	C = 221	O = 12	E = 3.75	R = 3.20	0.0003	0.0124
Biological process	B cell homeostasis	GO:000178	C = 9	O = 3	E = 0.15	R = 19.66	0.0004	0.0151
Biological process	Adaptive immune response based on somatic recombinationof immune receptors built from immunoglobulin superfamily domains	GO:000246	C = 59	O = 6	E = 1.00	R = 6.00	0.0004	0.0151
Biological process	Antigen processing and presentation of exogenous peptide antigen	GO:000247	C = 10	O = 3	E = 0.17	R = 17.69	0.0005	0.0175
Biological process	Response to lipid	GO:003399	C = 342	O = 15	E = 5.80	R = 2.59	0.0005	0.0175
Biological process	Adaptive immune response	GO:000225	C = 62	O = 6	E = 1.05	R = 5.71	0.0006	0.0202
Biological process	Negative regulation of mature B cell apoptotic process	GO:000290	C = 3	O = 2	E = 0.05	R = 39.31	0.0008	0.0234
Biological process	Mature B cell apoptotic process	GO:000290	C = 3	O = 2	E = 0.05	R = 39.31	0.0008	0.0234
Biological process	Regulation of mature B cell apoptotic process	GO:000290	C = 3	O = 2	E = 0.05	R = 39.31	0.0008	0.0234
Biological process	Activation of immune response	GO:000225	C = 66	O = 6	E = 1.12	R = 5.36	0.0008	0.0234
Biological process	Epidermis development	GO:000854	C = 92	O = 7	E = 1.56	R = 4.49	0.0009	0.0255
Biological process	Positive regulation of immune system process	GO:000268	C = 153	O = 9	E = 2.59	R = 3.47	0.001	0.0267
Biological process	Innate immune response	GO:004508	C = 94	O = 7	E = 1.59	R = 4.39	0.001	0.0267
Biological process	Antigen processing and presentation of peptide antigen	GO:004800	C = 13	O = 3	E = 0.22	R = 13.61	0.0012	0.0294

Biological process	Antigen processing and presentation of exogenous antigen	GO:001988	C = 13	O = 3	E = 0.22	R = 13.61	0.0012	0.0294
Biological process	Leukocyte mediated immunity	GO:000244	C = 71	O = 6	E = 1.20	R = 4.98	0.0012	0.0294
Biological process	Regulation of fibroblast proliferation	GO:004814	C = 29	O = 4	E = 0.49	R = 8.13	0.0013	0.0311
Biological process	Multicellular organismal process	GO:003250	C = 1895	O = 46	E = 32.14	R = 1.43	0.0017	0.0322
Biological process	Negative regulation of B cell apoptotic process	GO:000290	C = 4	O = 2	E = 0.07	R = 29.48	0.0017	0.0322
Molecular function	Molecular transducer activity	GO:006008	C = 309	O = 14	E = 4.99	R = 2.81	0.0004	0.029
Molecular function	Signal transducer activity	GO:000487	C = 309	O = 14	E = 4.99	R = 2.81	0.0004	0.029
KEGG pathway	Staphylococcus aureus infection		C = 15	O = 6	E = 0.21	R = 28.48	2.97^−08^	9.80^−07^
KEGG pathway	Complement and coagulation cascades		C = 17	O = 6	E = 0.24	R = 25.13	7.19^−08^	1.19^−06^
KEGG pathway	Systemic lupus erythematosus		C = 25	O = 5	E = 0.35	R = 14.24	2.10^−05^	0.0002
KEGG pathway	p53 signaling pathway		C = 25	O = 3	E = 0.35	R = 8.55	0.0049	0.027
KEGG pathway	Antigen processing and presentation		C = 25	O = 3	E = 0.35	R = 8.55	0.0049	0.027
KEGG pathway	Pathways in cancer		C = 142	O = 7	E = 1.99	R = 3.51	0.0037	0.027
KEGG pathway	MAPK signaling pathway		C = 123	O = 6	E = 1.73	R = 3.47	0.0075	0.0354
KEGG pathway	Pancreatic cancer		C = 34	O = 3	E = 0.48	R = 6.28	0.0117	0.0429
KEGG pathway	Colorectal cancer		C = 33	O = 3	E = 0.46	R = 6.47	0.0108	0.0429
KEGG pathway	Lysosome		C = 70	O = 4	E = 0.98	R = 4.07	0.0165	0.0495
KEGG pathway	Neuroactive ligand-receptor interaction		C = 72	O = 4	E = 1.01	R = 3.96	0.0182	0.0495
KEGG pathway	Chronic myeloid leukemia		C = 38	O = 3	E = 0.53	R = 5.62	0.0159	0.0495
KEGG pathway	Natural killer cell-mediated cytotoxicity		C = 41	O = 3	E = 0.58	R = 5.21	0.0195	0.0495

**Table 9.** GO and KEGG enrichment results for the 29 predicted and anticorrelated mRNAs targets of five miRNAs differentially expressed in the chronic period

GO terms	Term description	GO ID	Size of term	miRNA target	Expected	Enrichment ratio	Raw *p* value	FDR
Biological process	Negative regulation of DC differentiation	GO:2001198	C = 2	O = 2	E = 0	R = 576.79	2.88E-06	0.0005
Biological process	Regulation of DC differentiation	GO:2001199	C = 2	O = 2	E = 0	R = 576.79	2.88E-06	0.0005
Biological process	DC differentiation	GO:0097028	C = 13	O = 2	E = 0.02	R = 88.74	0.0002	0.0178
Biological process	Glial cell development	GO:0021782	C = 69	O = 3	E = 0.12	R = 25.08	0.0002	0.0178
Biological process	Regulation of glial cell proliferation	GO:0060251	C = 16	O = 2	E = 0.03	R = 72.10	0.0003	0.0214
Biological process	Glial cell proliferation	GO:0014009	C = 23	O = 2	E = 0.04	R = 50.16	0.0007	0.0356
Biological process	Regulation of immune system process	GO:0002682	C = 653	O = 6	E = 1.13	R = 5.30	0.0007	0.0356
Biological process	Response to wounding	GO:0009611	C = 692	O = 6	E = 1.20	R = 5	0.0009	0.0401
Biological process	Negative regulation of DNA binding	GO:0043392	C = 31	O = 2	E = 0.05	R = 37.21	0.0013	0.0498
Biological process	Oligodendrocyte development	GO:0014003	C = 32	O = 2	E = 0.06	R = 36.05	0.0014	0.0498
KEGG pathway	Hematopoietic cell lineage		C = 124	O = 3	E = 0.07	R = 40.29	5.70E-05	0.001
KEGG pathway	Lysosome		C = 79	O = 2	E = 0.05	R = 42.16	0.001	0.0015

Target genes that directly correlated with differentially expressed miRNAs during the epileptogenesis period were enriched for GO terms related to glia proliferation [“regulation of glial cell proliferation” (*p* = 0.0001) and “glial cell proliferation” (*p* = 0.0001)]. In addition, and as in the inverse correlation analysis, terms related to complement activation [like “complement activation, classical pathway” (*p* = 0.0004)] were significantly enriched. In the chronic period, the positively correlated mRNA targets revealed significant (*p* < 0.00001) enrichment in GO terms related to receptor function like “receptor activity,” “signaling receptor activity,” “G protein-coupled receptor activity,” “signal transducer activity,” “molecular transducer activity,” and “transmembrane signaling receptor activity.”

To infer the functional relationships between miRNAs identified as differentially expressed in the meta-analysis, we created a network of miRNAs based on their predicted anticorrelated target pathways (Fig. 5). These results highlight that several distinct miRNAs may contribute to the regulation of functionally related processes and pathways, and so prioritizing individual miRNAs as potential therapeutic targets will require downstream experimental analysis.

## Discussion

### Main findings

The present meta-analysis provides a miRNA differential expression signature in the DG of rats during epileptogenesis and in the chronic phase of epilepsy. We identified 26 miRNAs significantly differentially expressed during epileptogenesis, and five miRNAs significantly differentially expressed in the chronic phase of epilepsy. We also identified 11 miRNAs in epileptogenesis and two in chronic epilepsy that were identified as significantly differentially expressed by the meta-analysis but not in any of the individual studies. Further, we explored the negative correlation between the significantly differentially expressed miRNAs and their predicted mRNA targets in the same models of epilepsy. We identified 122 predicted mRNAs targets with an anticorrelated expression relationship to 22 of the 26 miRNAs significantly differentially expressed in epileptogenesis. Below, we discuss these findings and their possible implications in the development and maintenance of epilepsy. Together, we also discuss the intrinsic limitations of this study that must be taken into account.

### Epileptogenesis

Functional annotations of the target genes of miRNAs significantly differentially expressed during the latent interval between brain injury and the development of spontaneous seizures (epilepsy) support a relationship between dysregulated miRNAs and molecular and cellular reorganizations that are known to occur during epileptogenesis. First, the GO enrichment analysis identifies many terms that suggest a role for modulation of synaptic transmission during epileptogenesis. This is not surprising, given the critical role of the DG in the temporal lobe seizure network (Krook-Magnuson et al., 2015) and previous experimental evidence for changes in synaptic efficacy and connections during epileptogenesis (Dudek and Sutula, 2007). Another set of terms broadly refers to immunity and inflammation, events that are deeply associated with epileptogenesis (Vezzani et al., 2015). This is also supported by the identification of individual differentially expressed miRNAs (e.g., miR-146a-5p; Aronica et al., 2010) and mRNA targets (e.g., *CD74* and *C1r*; Teo and Wong, 2010; Zeis et al., 2016) involved in inflammation.

Worthy of note is the enrichment for genes in the MAPK signaling pathway. Enrichment within the MAPK cascade has been reported during latency in the pilocarpine model in a hippocampal RNA expression study based on high-throughput RNA sequencing (Hansen et al., 2014). In particular, we found robust upregulation of two MAPKs, *Map3k14*, also called NIK, and *Map3k4*, also called MEKK4. *Map3k14*, a target of miR-7a-5p and miR-138-5p, mediates the neuron specific suppression of the nuclear factor κ-B (*NF-kB*; Mao et al, 2016) that is upregulated in epilepsy patients (Teocchi et al., 2013) and in an experimental model of traumatic brain injury (Lipponen et al., 2016). *NF-kB* has been linked to traumatic brain injury relevant outcomes, including epileptogenesis and tissue repair, the hypothesis being that it plays an antiepileptogenic role (Lipponen et al., 2016). Thus, *Map3k14* activation may favor epileptogenesis and damage. The transcript of *Map3k4*, the other upregulated MAPK, is a target of four significantly downregulated miRNAs (namely, miR-92b-3p, miR-153-3p, miR-101a-3p, and miR-3573-3p). This enzyme activates the p38 and JNK pathways that are known to contribute to the apoptotic and inflammatory responses after kainate injection in mice (Yang et al., 1997; Jeon et al., 2000).

Activation of each of these suggested proepileptogenic kinase pathways might be counterbalanced by upregulation of inhibitors such as phosphatases. In our analysis, the protein tyrosine phosphatase, nonreceptor type 5 (*Ptpn5*), also called *STEP*, was found upregulated, and anticorrelated with the downregulated miR-150-5p and miR-383-5p. Contrary to *Map3k4*, *Ptpn5* has been shown to inhibit p38 by selectively dephosphorylating its activation loop tyrosines and by sequestering it in the cytosol (Francis et al., 2014). *Ptpn5* can also target the glutamate receptor subunits GluN2b and GluA2 leading to receptor internalization and decreased synaptic efficiency (Snyder et al., 2005; Xu et al., 2009). In addition, *Ptpn5* inhibits the ERK2 pathway. Whereas p38 downstream molecules lead to the activation of neuroinflammation and apoptotic processes, the ERK2 cascade triggers neuronal differentiation and survival through the activation of the antiapoptotic gene *bcl-2* (Cruz and Cruz, 2007).

In addition to above, miRNAs such as miR-101a-3p, miR-551b-3p, and miR-139-5p may act together to modulate the expression of the predicted target *syn2*, a gene that is mutated in epileptic patients (Cavalleri et al., 2007). Synapsin 2 is a member of the synapsin family composed by synaptic vesicle phosphoproteins that modulate synaptic transmission and plasticity. Notably, Syn2-knock-out mice show a decreased vesicle density at inhibitory synapses of DG GCs and are prone to epileptic seizures (Medrihan et al., 2013). Here, we found an inverse correlation of three downregulated miRNAs (miR-101a-3p, miR-551b-3p, and miR-139-5p) with the Syn2 mRNA, which levels are slightly increased suggesting that Syn2 phophoproteins, at this stage (i.e., epileptogenesis), are still able to control neuronal transmission at the DG synapses. Notably, Syn2 interacts with presynaptic Ca^2+^ channels to promote GABA asynchronous release (Medrihan et al., 2013), maintaining the tonic inhibition of excitatory neurons and contrasting the aberrant network synchronization that lead to seizures development in the chronic phase. These findings suggest an antiepileptogenic role of this inverse-correlation. The dentate cells, in this case, may slow down the miRNA levels to contrast the upcoming epileptogenic process.

High levels of miR-212-5p may favor the epileptogenic process through reduced expression of *Gabrd*. We found that the expression of the subunit δ of GABA_A_ receptors was decreased in DG and inverse-correlated with the upregulated miR-212-5p. δ-Subunit-containing receptors are found in extrasynaptic and perisynaptic locations in hippocampal DG GCs (Wei et al., 2003). Because of their high affinity for GABA, they mediate tonic GABA_A_ inhibition (Stell et al., 2003). Therefore, a decrease in GABA_A_ receptor δ-subunits may impair tonic GABA inhibition, contributing to GCs hyperexcitation and seizures onset.

### Chronic epilepsy

Our exploration of the chronic stage of epilepsy was more limited than that for epileptogenesis as anticorrelations (inferred via statistically significant gene and miRNA fold changes in response to the disease) with miRNA targets could be evaluated only for the amygdala stimulation model. This analysis highlighted the downregulation of five miRNAs and the upregulation of several mRNAs targets, and identified genes enriched in GO terms related to glial cells and dendritic cells (DCs). Whereas the proliferation of glia cells and their contribution to neuroinflammation and hyperexcitability in chronic epilepsy are well recognized (Devinsky et al., 2013; Robel and Sontheimer, 2016), the role of DCs in the context of epilepsy remains elusive. It can be hypothesized that DCs might be involved in epilepsy by maintaining a chronic inflammatory response (Ludewig et al., 2016).

Among the list of mRNAs predicted targets anticorrelated with significantly differentially expressed miRNAs in the chronic stage of epilepsy, of note is the glutamate ionotropic receptor δ type 2 (*Grid2*), which is anticorrelated with miR-130a-3p, miR-148b-3p, and miR-551b-3p. The involvement of ionotropic glutamate receptors in epileptic hyperexcitability is well established, but not much is known specifically on glutamate ionotropic type δ receptors or the regulation of this process. Further studies are needed to establish a role of these receptors in hippocampus and more specifically in epilepsy.

### Limitations

The purpose (and, in our view, the strength) of this work was to maximize information from underpowered individual studies, increasing power and allowing the identification of a set of miRNAs that, being significantly and similarly dys-regulated in multiple experimental models, may be related to the disease rather than specific to a particular model. The study, however, also has limitations that should be taken into account.

A technical limitation is that the comparison of datasets in which tissue was obtained through different methods and that used different microarray platforms may have led to some miRNAs being detected in one experimental model and not in another, due to technical differences related to the assay system. Therefore, we cannot exclude the possibility that additional miRNAs were significantly dys-regulated.

Other limitations refer to biological aspects. First, miRNAs are only one mechanism of regulation of gene expression. Other changes may occur depending on other epigenetic mechanisms (histone modifications, DNA methylations) or changes in transcription factors. Second, this analysis has been conducted on one specific hippocampal subarea, the DG, enriched in a specific cell population, the GCs. Other brain areas and cell populations may be equally or even more important in epileptogenesis. Third, due to limitation in the availability of datasets, we analyzed data from a single time point in all models, but epileptogenesis may develop differently in different models. Finally, given the lack of miRNA and mRNA datasets in epileptic patients matched with valid controls, we could not verify whether the miRNA-mRNA interactions identified in rats may be relevant for the human disease.

In addition, the comparison with mRNA datasets should be viewed as a secondary outcome of the study and considered with caution. In this study, this comparison is primarily based on the assumption that miRNAs decrease levels of their mRNA targets (Guo et al., 2010) and, therefore, that target mRNAs will undergo changes in anticorrelation with those of miRNAs. Although this is the best characterized mechanism of miRNA action, it is becoming evident that miRNAs also have specific nuclear functions, including transcriptional control of gene expression and regulation of alternative splicing (Catalanotto et al., 2016), which may not lead to anticorrelation between miRNA and mRNA levels. Therefore, we also performed a further analysis of direct correlation between miRNAs and mRNAs. These analyses now require verification and further studies to establish the exact patterns of interaction between miRNAs and mRNAs in the epileptic tissue and their functional impact on epileptogenesis and maintenance of an epileptic condition.

## Conclusions

The present meta-analysis identified many significantly differentially expressed miRNAs in epileptogenesis and chronic epilepsy, several of which were not uncovered in the individual studies, highlighting the additional information that can be gained by meta-analysis. Our results also highlight the added value of meta-analysis of existing data and so avoid unnecessary animal experimentation to generate new hypotheses on miRNAs involved in epileptogenesis and chronic epilepsy. Our results highlight a possible key role for a few miRNAs that are worthy of further investigation. As it may be expected, however, the number and heterogeneity of mRNAs identified by this meta-analysis suggest that therapies focused on a single miRNA target may be not sufficient to reverse or ameliorate the epileptogenic process.

## Synthesis

Reviewing Editor: Alfonso Represa, INSERM Decisions are customarily a result of the Reviewing Editor and the peer reviewers coming together and discussing their recommendations until a consensus is reached. When revisions are invited, a fact-based synthesis statement explaining their decision and outlining what is needed to prepare a revision will be listed below. The following reviewer(s) agreed to reveal their identity: Katja Kobow, Gary Brennan

Referees evaluated your report and appreciated that most of their concerns and suggestions have been addressed. However, there are two points that you did not apparently included in your resubmission:

1. Analyses whether miR expression correlated to some extent with up-regulated gene expression

2. Whether the genes showing a direct correlation were implicated in other (additional) pathways than the down-regulated ones.

We think that these points are not difficult to evaluate and that they would improve the output of the study.

In addition to this, we feel that the referencing needs to be revised. There is an over-reliance on review articles and specific authors and it does not represent the current field and state of the field.

Another minor comment concerns the general abundance of the novel microRNAs identified in the manuscript. A 2-fold change on microRNAs would have different impact on DG cells depending on its physiological expression levels, whether too low or too high.
